# Structured interconnectivity optimizes neural geometry for balancing specificity and generalization in object recognition

**DOI:** 10.1038/s42003-025-09411-y

**Published:** 2025-12-27

**Authors:** Yiyuan Zhang, Jirui Liu, Jia Liu

**Affiliations:** Tsinghua Laboratory of Brain and Intelligence, Department of Psychological and Cognitive Sciences, Tsinghua University, Beijing, China

**Keywords:** Object vision, Neural encoding

## Abstract

Balancing specificity and generalization in object recognition is a significant challenge for biological and artificial visual systems. Here, we investigated how the brain addresses this challenge by examining the relationship between interconnectivity of neural networks, dimensionality of neural space, and levels of abstraction in representing objects, employing combined neurophysiological data from macaques and computational modeling. We found that higher interconnectivity within area TEa of macaques’ inferior temporal (IT) cortex was associated with lower dimensionality and greater generalization, while lower interconnectivity within area TEO correlated with higher dimensionality and greater specificity. To establish a causal link, we developed a brain-inspired computational model constrained by empirical wiring length. This structured interconnectivity created optimal dimensionality of the neural space, facilitating efficient energy distribution across the representational manifold embedded within the neural space, balancing specificity and generalization. Our findings underscore the critical role of structured connectivity in enabling robust object recognition through multi-level abstraction.

## Abstract (web summary)

Mechanistic modeling of macaque IT cortex reveals how structured interconnectivity optimizes neural geometry to balance specificity and generalization in object recognition.

## Introduction

Consider the task of recognizing different dog breeds, such as Dalmatians and Labradors. Specificity focuses on the unique features, such as the distinctive spots and graceful movements of Dalmatians and the sleek body and friendly eyes of Labradors, while generalization does the opposite, emphasizing common characteristics and overlooking these distinct differences. The challenge in object recognition lies in balancing these processes: differentiating the intricate details that set them apart while simultaneously capturing the broader features that generalize them into the single category of “dog”^1–5^. Computational modeling suggests that specificity and generalization can be achieved through linear (e.g., weighted summation) operations to build unique features and non-linear (e.g., thresholding or max-pooling) operations to increase generalization across transformations^2,4,6–10^ along a processing hierarchy (e.g., goal-driven hierarchical convolutional neural networks), where a sequence of computations from stacks of layers generate invariant object recognition behavior^7,11–16^.

Recent advances in population coding provide a new perspective on how the brain achieves multiple levels of abstraction^2,4,17^. In a high-dimensional neural space constructed by a population of neurons, objects sharing the same features form a neural manifold^18–20^. The high dimensionality of this manifold allows for rich and detailed encoding of distinct features, enhancing the ability to distinguish between similar objects through sparse coding and fine-grained representation^20–22^. Conversely, the low dimensionality focuses on the most salient and common features shared across different instances of an object category, making the representation robust to variations and thus aiding in quick and efficient categorization^23,24^. In this framework, we suggest that a visual system can achieve the balance between specificity and generalization by maintaining an intermediate level of dimensionality. This intermediate dimensionality allows the system to capture sufficient details for distinguishing individual objects while being abstract enough to recognize broader categories. Thus, by avoiding the pitfalls of overly detailed or overly simplified representations, an intermediate dimensionality ensures that the neural coding is both versatile and efficient.

Previous studies have shown that structural connections play a crucial role in shaping dimensions of neural manifolds^25–30^. One structural factor that influences the dimensionality is anatomical interconnectivity among neurons^31,32^. High interconnectivity allows neurons to be densely connected, enabling shared features to be easily combined across different objects. This facilitates the creation of more generalized and abstract representations^33,34^, ultimately reducing the overall dimensionality of the neural manifold^35,36^. Conversely, low interconnectivity leads to neurons that are more specialized and less influenced by other neurons^37,38^, thereby allowing for a higher-dimensional representation, with each dimension encoding specific and unique features of the object^39^. Thus, to achieve an effective balance between specificity and generalization, we suggest that the neural system might employ an optimal level of anatomical interconnectivity.

**Fig. 1:**
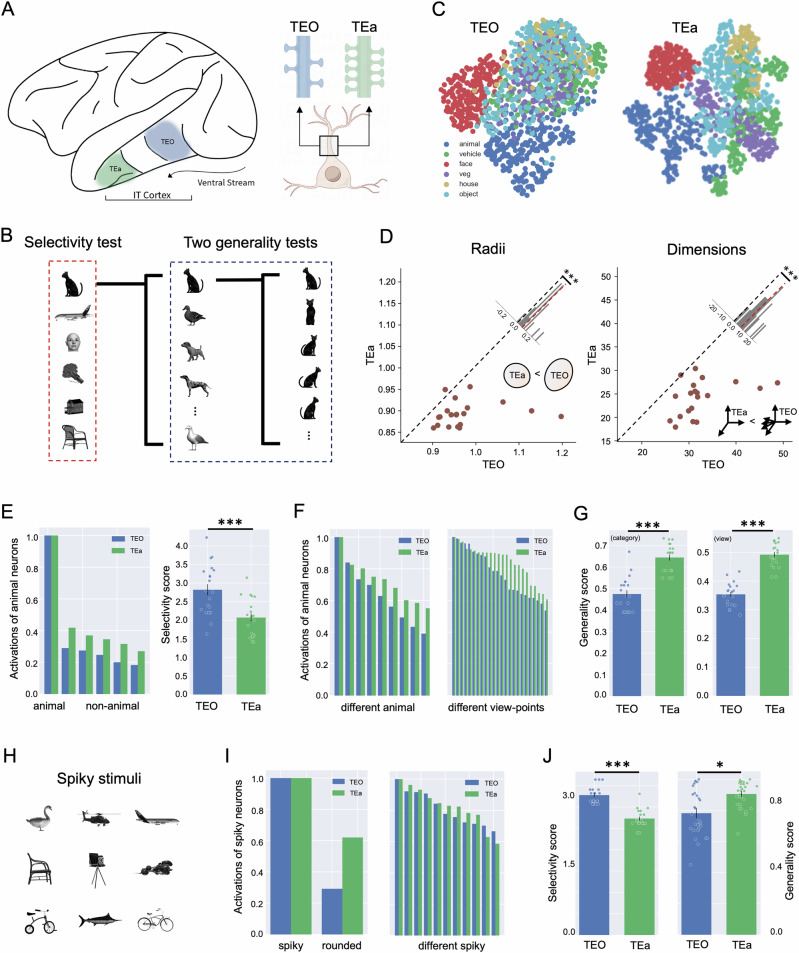
The gradient of specificity and generalization along macaques’ IT cortex. **A** Left: Schematic illustration of the IT cortex, including the TEO and TEa. Right: Schematic illustration of dendritic spines on the dendrites of neurons in the TEO and TEa. Schematic illustrations were created by FigDraw (www.figdraw.com). **B** During the recording, macaques passively viewed stimuli from six object categories: animals, vehicles, faces, vegetables, houses, and tools. Each exemplar consisted of 24 different views. **C** Neural manifolds of stimuli visualized in a 2D subspace using UMAP. Each point represents the neural state of one stimulus, with a total of 1224 neural states. Different colors represent different object categories. **D** Radii and dimensions of neural manifolds formed by neurons in the TEO and TEa. Diagonal lines indicate equal radii (left) and dimensions (right). Points below the diagonal lines indicate higher dimensions and larger radii of the TEO compared to the TEa. Each point is the result of a permutation test, randomly sampling 100 neurons from the neuron pools in TEO and TEa, respectively. Histograms in the upper right show the distribution of radii and dimensions. **E** Specificity. Response magnitudes of animal-responsive neurons in the TEO and TEa to six object categories (left) and corresponding specificity scores (right). **F** Generalization. Response magnitudes of animal-responsive neurons in the TEO and TEa to various animal exemplars (left) and different views of the same exemplar (right). **G** Generalization scores of the TEO and TEa in generalizing across different animal exemplars (left) and different views (right). **H** Exemplars of spiky stimuli. **I** Response magnitudes of spiky-responsive neurons in the TEO and TEa to spiky versus non-spiky stimuli (specificity, left) and to different types of spiky stimuli (generalization, right). **J** Specificity and generalization scores of the TEO and TEa in differentiating spiky versus non-spiky stimuli (left) and in generalizing across different spiky stimuli (right). ***p < 0.001; *p < 0.05.

To test our conjecture that the balance of specificity and generalization can be achieved by maintaining an intermediate level of dimensionality through an optimal level of interconnectivity, we analyzed neurons in the TEO and TEa regions of the inferior temporal (IT) cortex in macaques (Fig. 1A) recorded previously by Bao et al.^39^ when the macaques passively viewed a series of objects from different categories and viewing angles (Fig. 1B). We chose the TEO and TEa as the regions of interest for two main reasons. First, abundant evidence indicates that these regions are central to object recognition, possessing both specificity and generalization^3,4,40–44^. Second, previous anatomical studies of the IT cortex have shown a gradient in dendritic spine density from the posterior to the anterior IT cortex^45–47^. Specifically, neurons in the TEa possess significantly higher dendritic spine counts (approximately 11,000 per neuron) compared to those in the TEO (about 5000) (Fig. 1A). Higher dendritic spine density suggests a higher degree of anatomical connections to other neurons, indicating higher interconnectivity^48,49^. Therefore, we predicted that the TEa, with its higher interconnectivity, constructs a neural representational space with lower dimensionality, which in turn results in lower specificity but higher generalization, as compared to the TEO with lower interconnectivity.

## Results

### Gradients of interconnectivity, abstraction, and dimensionality in IT cortex

A total of 307 neurons were recorded from the TEO (106 neurons) and TEa (201 neurons) in response to six object categories: animals, vehicles, faces, vegetables, houses, and tools. Each category contains 4 to 11 exemplars, each viewed from 24 different views, which made a total of 1224 stimuli (Fig. 1B). To visualize neural manifolds of these object categories formed by TEO and TEa neurons, we utilized the Uniform Manifold Approximation and Projection (UMAP) method^50^ to project the high-dimensional neural states of all object exemplars into a 2D subspace (Fig. 1C). Visual inspection of the UMAP projection revealed that in the TEa, neural states of object exemplars were more tightly clustered within each point cloud and more dispersed between them compared to those in the TEO. To quantify this observation, we utilized a method developed by Chung et al.^19^ to calculate the effective dimensions of the manifolds and the radii of point clouds formed by neurons in the TEO and TEa, respectively. Consistent with the visual inspection, the radius of each point cloud in the TEa was significantly smaller than that in the TEO (*p* < 0.001) (Fig. 1D, left). Critically, the effective dimensionality of the neural manifold in the TEa was significantly lower than that in the TEO (24.19 versus 32.40; *t*(38) = 5.31, *p* < 0.001) (Fig. 1D, right). This suggests that the TEa, with its higher interconnectivity, constructs a neural representational space with lower dimensionality, embedding the neural states of object categories in more clustered point clouds with smaller radii. Taken together, we observed a gradient of decreased dimensionality along the IT cortex, accompanied by increased interconnectivity.

To explore the gradient of abstraction along the IT cortex, we examined the specificity and generalization of these two regions. Among all recorded neurons, 16.9% showed selective responses to a single object category. Consistent with criteria established in previous studies^33^, we defined object-responsiveness as a neuron’s average response magnitude to one object category being at least twice the maximum response magnitude observed across the remaining categories. Notably, most of these neurons (78.8%) were specifically tuned to the animal category (Table S1). Accordingly, we focused on the specificity and generalization of the TEO and TEa neurons in response to the animal category. For visual comparison, we ranked the response magnitudes of animal-responsive neurons to animals and non-animal objects in descending order, normalizing the highest responses (i.e., to animals) to 1 (Fig. 1E, left). The level of specificity was indexed by the ratio of the average response magnitude to animals to the maximum response magnitude to non-animal objects (see “Materials and methods”). We found that animal-responsive neurons in the TEO exhibited relatively lower responses to non-animal objects compared to those in the TEa (Fig. 1E, left), indicating a higher level of specificity in the TEO (*t*(38) = 4.46, *p* < 0.001) (Fig. 1E, right).

A similar analysis was conducted to examine the generalization by classifying nine animal exemplars, each consisting of 24 different views, into basic-level animal categories (i.e., 2 dogs, 1 cat, 2 horses, 3 birds, 1 duck) (Fig. 1B). Figure 1F (Left) shows the magnitudes of animal-responsive neurons to different animal exemplars ranked in descending order, with the highest response normalized to 1. The level of generalization was indexed by the ratio of the minimum to maximum magnitudes of the animal-responsive neurons to different animal exemplars (see “Materials and methods”). We found that neurons in the TEa exhibited higher responses to most animal exemplars compared to those in the TEO, indicating a significantly higher degree of generalization in the TEa (*t*(38) = −5.79, *p* < 0.001) (Fig. 1G, left). A similar pattern was found for the generalization across different views within exemplars (Fig. 1F, right), indicating that neurons in the TEa showed a significantly higher level of generalization across views compared to those in the TEO (*t*(38) = −11.18, *p* < 0.001; Fig. 1G, right). Another index to measure generalization is standard deviation, with a lower level of standard deviation among neural responses to animal exemplars indicating a higher level of generalization. We found similar results using this index (Fig. S1).

To verify that the observed specificity–generalization gradient along the IT cortex was not an artifact of our neuron-selection criterion, we conducted a sensitivity analysis by systematically varying selection thresholds. For each threshold, we re‑estimated the indices of between-category specificity and within‑category generalization. Across varying thresholds, the TEO exhibited higher specificity, whereas the TEa exhibited greater generalization across exemplars and views (Fig. S2). This sensitivity analysis confirms the threshold-independence and robustness of the specificity–generalization gradient.

To further substantiate the findings obtained using univariate metrics based on the response magnitude ratios, we performed multivariate representational similarity analysis (RSA) comparing neuronal populations in areas TEO and TEa. Specifically, we computed representational similarity matrices (RSMs) to quantitatively evaluate between-category specificity and within-category generalization across exemplars and viewing angles. Mean representational similarity values derived from these matrices provided indices of representational distinguishability. Consistent with the univariate findings, the multivariate analyses confirmed significantly lower between-category similarities in area TEO, indicative of higher representational specificity compared to TEa (TEO vs. TEa: 0.56 vs. 0.66; *t*(38) = −5.86, *p* < 0.001). In contrast, significantly higher within-category similarity was observed in area TEa, demonstrating enhanced generalization at both the exemplar level (TEO vs. TEa: 0.68 vs. 0.81; *t*(38) = −13.60, *p* < 0.001) and the view level (TEO vs. TEa: 0.74 vs. 0.87; *t*(38) = −57.42, *p* < 0.001; Fig. S3). In sum, along the gradient of increased interconnectivity, we observed a gradient of increased abstraction from specificity to generalization along the IT cortex.

Additionally, we examined the specificity and generalization in the TEp, whose neurons exhibit a comparable number of dendritic spines to those in the TEO (i.e., a similar level of interconnectivity) but receive inputs more similar to those of the TEa^45–47,51^. We found that the pattern of dimensions and abstraction in the TEp was more similar to that of the TEO (Fig. S4), supporting the conjecture that interconnectivity plays a critical role in influencing specificity and generalization in object recognition.

The initial analysis focused on categorizing objects into natural categories, such as animate versus inanimate. Here, we further examined whether the gradient of increased abstraction is also applicable to specific object features across different object categories. Specifically, we re-categorized objects based on the presence or absence of protrusions, a feature dimension previously identified by Bao et al.^39^, labeling them as either spiky (objects with protrusions, Fig. 1H) or non-spiky. In the TEO and TEa, 46.58% of neurons showed a higher response to spiky objects, whereas only 4.23% showed the opposite response pattern. Accordingly, we selected spiky-responsive neurons for further analysis. We found that the spiky-responsive neurons in the TEO exhibited a higher level of specificity (*t*(38) = 6.25, *p* < 0.001), whereas the TEa showed a higher level of generalization (*t*(38) = −2.56, *p* < 0.05) (Fig. 1I, J). Thus, the differential preference for specificity and generalization observed in the TEO and TEa extends to specific object features, not just natural object categories, indicating that the gradient of increased abstraction along the ventral pathway is a general property of the IT cortex, applicable across various object recognition contexts. In addition, we conducted this analysis for each monkey separately and replicated the main findings in both monkeys, showing that the TEO exhibited significantly higher dimensions, larger radii, higher specificity, and lower generalization compared to TEa (Figs. S5 and S6).

In summary, neurons in the TEa, which possess a higher level of anatomical interconnectivity and thus lower dimensionality, compromised their specificity to their preferred object category to enhance their proficiency in generalizing across different exemplars of the same category compared to those in the TEO. This relationship between interconnectivity and abstraction supports our conjecture that interconnectivity modulates the dimensionality of neural space, which subsequently influences abstraction. However, there are three unresolved issues. First, the relationship between the density of dendritic spines and the degree of interconnectivity is indirect. Additionally, dendritic spine density primarily reflects excitatory synapses^52^ and thus does not adequately capture the crucial role played by inhibitory circuits. Second, the link between interconnectivity and dimensionality is only associative but not causal. Finally, the mechanism by which dimensionality modulates the abstraction remains unclear. To address these issues, we next constructed a brain-inspired neural network to first replicate the same capability of IT neurons for object recognition and then establish a causal link among interconnectivity, dimensionality, and abstraction, to elucidate underlying mechanisms.

### Brain-inspired neural model to link interconnectivity to functionality

To examine the causal role of anatomical interconnectivity in shaping representational geometry, we implemented a computational model based on the human ventral temporal cortex (VTC) rather than Macaque IT cortex. Human VTC offers the advantage of extensively characterized fine- and large-scale functional maps from numerous fMRI studies examining representational specificity and generalization in object recognition^53–58^. Moreover, prior computational work has developed two-dimensional self-organized maps (SOMs) to simulate functional organization of human VTC^59^. This spatially explicit cortical-sheet implementation allows systematic manipulation of lateral interconnectivity by employing biologically realistic wiring length derived from empirical brain data. Besides, the well-established functional homology between human VTC and macaque IT cortex supports using this human VTC-based model to test representational principles suggested by macaque neurophysiological recordings^17^.

**Fig. 2:**
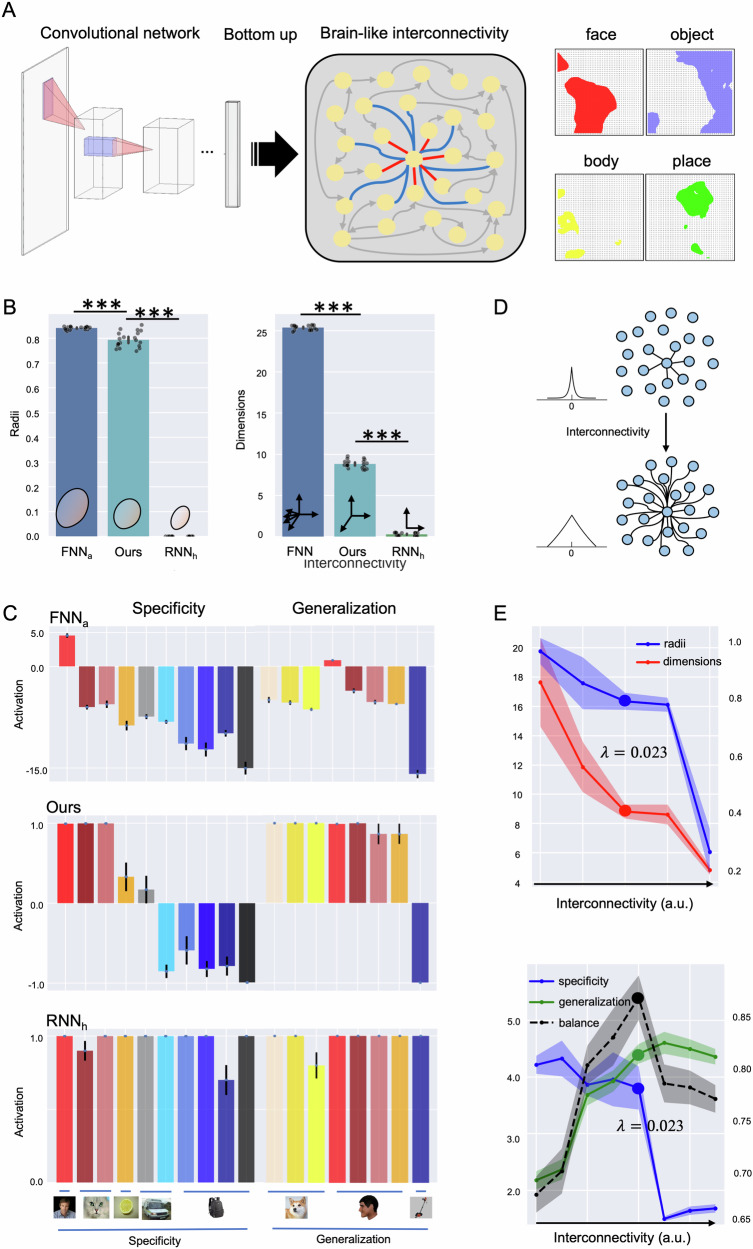
The brain-like computational model. **A** Left: The architecture of the model comprises two main parts: a DCNN encoder, which translates images into vector representations (i.e., object space), and a 2D lattice of SOM with physical lateral connections to simulate human VTC. Right: The SOM lattice was organized into category-responsive clusters for faces, daily objects, bodies, and places, mirroring the functional specialization observed in the VTC. **B** Radii and dimensions of representational manifolds in three types of neural networks (NN): feedforward NN (FNN_a_), our model, and recurrent NN (RNN_h_). **C** Specificity and generalization in object recognition. Stimuli used for testing specificity included human faces, faces from different species (dog and cat), objects with shared shapes (lemon), objects with shared configurations (ambulance and airplane), and familiar objects (store, backpack, pitcher, speaker). Stimuli for testing generalization included faces from different species (cat, dog, and tiger), human faces from different views (front, profile, cheek, back), and tools as a baseline. Detailed exemplars of these stimuli are provided in Fig. S7. Bar charts show the average activations of the networks with standard deviation. Note that the average activation of our model was derived from neurons in the face cluster after the model had stabilized. **D** Schematic illustration of the parameter $\lambda$ in the exponential distance rule (EDR), a constant that determines the range of lateral connections and thus modulates the interconnectivity of the network. The smaller the parameter $\lambda$, the wider the connections between neurons. **E** Top: Radii (blue) and dimensions (red) varied with different levels of interconnectivity in our model. Left y-axis: dimensions; right y-axis: radius sizes (a.u.). Bottom: Specificity (blue) and generalization (green) scores across different levels of interconnectivity ($\lambda$ = 0.5, 0.1, 0.075, 0.05, 0.023, 0.01, 0.0005, 0.0). Left y-axis: specificity scores; right y-axis: generalization scores; x-axis: wiring lengths $\lambda$ in a descending order. Shaded areas denote standard deviations. The black dashed lines represent the balance scores at different levels of interconnectivity, respectively. ***p < 0.001.

Specifically, the construction of this brain-inspired computational model involved four major steps (Fig. 2A). First, we implemented category-responsive regions in a two-dimensional lattice of a VTC-SOM model to simulate human VTC (Fig. 2A, left). Specifically, the VTC-SOM acquired a high-dimensional object space from AlexNet, a pre-trained deep convolutional neural network (DCNN) specifically designed for object recognition^60^, and then mapped it onto the 2D lattice (see “Materials and methods”). We then fed the stimuli from four categories (face, tools, body, and place) used in the fMRI study of the HCP dataset^61^ into this network to generate four distinct clusters, with neurons in each cluster tuned to one of the object categories (Fig. 2A, right). Previous studies have shown that this VTC-SOM model exhibits a topological hierarchy and functional specialization similar to those observed in the VTC^59^.

Second, we implemented the network’s anatomical interconnectivity by adding physical lateral connections among neurons in the lattice (i.e., intra-layer recurrence; for external recurrence, see Kubilius et al.^62^), which are typically absent in standard SOMs or DCNNs. These connections were based on the principle of wiring cost minimization that neuronal interconnections are predominantly local with some long-range connections^63,64^, encapsulated by the exponential distance rule (EDR)^65–67^:

$${\gamma }_{{ij}}=\left\{\begin{array}{c}\,1,\,w.p.{\,e}^{-\lambda {d}_{{ij}}}\\ 0,{otherwise}\end{array}\right.$$

where ${\gamma }_{{ij}}$ denotes the physical connection between neuron *i* and *j*, and ${d}_{{ij}}$ is the Euclidean distance between them in the lattice. The probability of a connection decreases as ${d}_{{ij}}$ increases. In the EDR, the most critical parameter is $\lambda$, a constant that determines the range of lateral connections. This parameter $\lambda$ controls how rapidly the probability of a connection decreases with distance, thus constraining the spatial extent of neuronal interactions within the network. Specifically, smaller $\lambda$ values correspond to broader, more extensive lateral connectivity, while larger $\lambda$ values restrict connectivity to more localized neighborhoods. In our model, since neighboring neurons typically share similar response preferences, restricting lateral connections to local neighborhoods is functionally equivalent to having sparser interconnectivity at the network level. Accordingly, the parameter $\lambda$ conceptually aligns with the anatomical gradient in dendritic spine density within IT cortex. That is, higher spine densities correspond to broader connectivity (smaller $\lambda$ values, characteristic of the TEa) and lower spine densities reflect sparser connectivity (larger $\lambda$ values, characteristic of the TEO). In the human temporal lobe, $\lambda$ is estimated to be approximately 0.1^65^. To calculate the corresponding $\lambda$ in the lattice, we utilized the Symmetric Normalization algorithm^68^ to warp the lattice of the SOM to the flattened human VTC in the Multi-Modal Parcellation template^69^, resulting in a model-corrected $\lambda$ of 0.023 (see “Materials and methods”). Therefore, $\lambda$ in our model reflects the biological constraint of wiring length in human VTC.

Third, we specified the functional component of interconnectivity using both Hebbian and anti-Hebbian rules^70–72^. The Hebbian rule strengthens excitatory connections between functionally similar neurons, whereas the anti-Hebbian rule establishes inhibitory connections between functionally different neurons:

$${w}_{{ij}}^{{hebb}}=\frac{1}{N}\mathop{\sum }\limits_{\mu }{x}_{i}^{\mu }{x}_{j}^{\mu }\,\left(\mu ={{\rm{face}}},{{\rm{object}}},{{\rm{body}}},{{\rm{place}}}\right)$$

where ${w}_{{ij}}^{{hebb}}$ represents the weight between neuron *i* and *j*, determined by their activation states within the *μ*th object-responsive cluster, and $N$ is the total number of neurons. In this formula, ${x}_{i}^{\mu }$ indicates the activity state of the *i*th neuron for the $\mu$ th cluster. For example, if neuron *i* is in the face cluster, then ${x}_{i}^{{face}}=1$, while ${x}_{i}^{{object}},{x}_{i}^{{body}},$ and ${x}_{i}^{{place}}$ are all set to $-1$. This configuration creates excitatory connections within the same cluster and inhibitory connections across different clusters. Accordingly, the connection weight ${w}_{{ij}}$ between neuron *i* and *j* is achieved by incorporating both spatial and functional components: ${w}_{{ij}}={\gamma }_{{ij}}\cdot {w}_{{ij}}^{{hebb}}$, which together determines the structure of network’s interconnectivity. Notably, within the anatomical constraints imposed by the wiring length parameter $\lambda$, Hebbian and anti‑Hebbian learning rules here organize synaptic weights by strengthening connections within categories and attenuating interactions between categories. These adjustments yield stable attractor basins corresponding to distinct object categories without modifying the underlying anatomical connectivity. Therefore, parameter $\lambda$ defines the anatomical connectivity scaffold, upon which Hebbian and anti-Hebbian learning rules refine intra- and inter-cluster connectivity, establish both excitatory and inhibitory connections while remaining within anatomically constrained limits.

Fourth, we incorporated temporal dynamics into the network to ensure continuous neural state updates (Fig. S9A), reflecting the stochastic nature of neural processing^71,73^. The probability of activation of neuron $i$ given condition ${b}_{i}$ is a sigmoid function weighted by $\beta$: $p({s}_{i}=+1|{b}_{i})=1/(1+{e}^{-\beta {b}_{i}})$. The magnitude of $\beta$ represents intrinsic noise in dynamics, which was set to 100 to simulate a very low-level noise.

In summary, by integrating physical lateral connections governed by EDR parameterized by $\lambda$, Hebbian and anti-Hebbian learning rules guided by functional similarity, and temporal dynamics, we constructed brain-like structured interconnectivity within the network. This approach greatly extends the empirical information available from brain data, while preserving the essential characteristic of the dendritic spine density observed in the IT cortex. Furthermore, by systematically changing the wiring length parameter $\lambda$, we can precisely manipulate the network’s level of interconnectivity. This manipulation enables us to examine how changes in the level of interconnectivity affect the dimensionality of the neural space, which in turn influences the abstraction of object representation.

To evaluate the performance of our model, we compared it with a standard feedforward neural network (e.g., AlexNet of DCNNs, FNN_a_), which lacks lateral connections among neurons, and a standard recurrent neural network (Hopfield Neural Network of RNNs, RNN_h_), characterized by fully interconnected neurons (see “Materials and methods”), as general baselines. Both feedforward and recurrent neural networks have been widely deployed in object recognition tasks^74,75^. As expected, the FNN_a_ exhibited the highest dimensionality and the largest radius of point clouds (Fig. 2B), indicating a more detailed and complex representation of object features. In contrast, the RNN_h_ displayed the lowest dimensionality and the smallest radius (Fig. 2B), reflecting a more compact and abstract representation. Our model showed intermediate values in both dimensionality and radius, suggesting a balanced representation of object features.

We then evaluated the networks’ ability to recognize faces, as faces have been extensively examined in human fMRI studies addressing the trade-off between specificity and generalization, thus offering abundant empirical data for comparison. Three types of representative stimuli were chosen for this evaluation. The first type included faces from different species (e.g., dogs’ and cats’ faces) and various view angles (e.g., profile, cheek) to assess the networks’ generalization. The second type included non-face objects that share shapes (e.g., lemons) or configurations (e.g., ambulances and airplanes), and the third type included daily objects as familiar as faces (e.g., tools, backpacks), both aimed at evaluating the networks’ specificity. The FNN_a_ showed extreme specificity to the human faces it was trained on, with minimal response to other face types or non-face objects (Fig. 2C, top). In contrast, the RNN_h_ showed extreme generalization, responding broadly to various faces and non-face objects (Fig. 2C, bottom). Our model, which incorporates structured interconnectivity, struck an ideal balance between specificity and generalization. Specifically, the face cluster in our model responded robustly to all tested face types (i.e., generalization) while showing limited response to non-face objects (Fig. 2C, middle). This balanced specificity and generalization mirrors findings from functional neuroimaging studies on the fusiform face area (FFA) in humans, named for its role in face perception^53^, and from neurophysiological studies on the middle face patch in macaques^33,76^.

Critically, to investigate the influence of the level of interconnectivity on face recognition, we systematically manipulated the parameter $\lambda$, which reflects wiring length between neurons (Fig. 2D). Specifically, we varied it to 0.0, 0.01, 0.05, and 0.1, either above or below the value typical of the human cortex ($\lambda$ = 0.023, model-corrected). We found a monotonic increase in both dimensionality and radii as interconnectivity decreased (dimensionality: one-way ANOVA, *F*(4,145) = 168.99, *p* < 0.001; radii: one-way ANOVA, *F*(4,145) = 382.92, *p* < 0.001) (Fig. 2E, top). These findings are consistent with observations from macaques’ IT cortex, where higher interconnectivity in the TEa resulted in a lower effective dimensionality and radius, suggesting that interconnectivity plays a causal role in modulating the geometric characteristics of neural manifolds.

In addition, we assessed our model’s specificity and generalization in face recognition by varying solely the parameter $\lambda$ (i.e., different levels of interconnectivity). As expected, the model’s specificity monotonically increased as interconnectivity decreased, whereas generalization showed the opposite trend, decreasing with lower interconnectivity (Fig. 2E, bottom). To further corroborate the findings obtained through univariate metrics, we performed a multivariate RSA on neural population responses within the computational model’s face-selective region. The results of these multivariate analyses closely aligned with the univariate findings, confirming that decreased interconnectivity (i.e., higher $\lambda$ values) significantly enhanced representational specificity while concurrently decreasing generalization (Fig. S8). Importantly, the model with a wiring length ($\lambda$ = 0.023) corresponding to the human cortical connectivity exhibited a near-optimal balance between representational specificity and generalization (Fig. 2E, bottom), implying that our brain is equipped with a neural architecture precisely calibrated for balancing these competing demands, necessitating a dedicated equilibrium between specificity and generalization for daily life object recognition tasks.

### Energy distribution across representation manifold

**Fig. 3:**
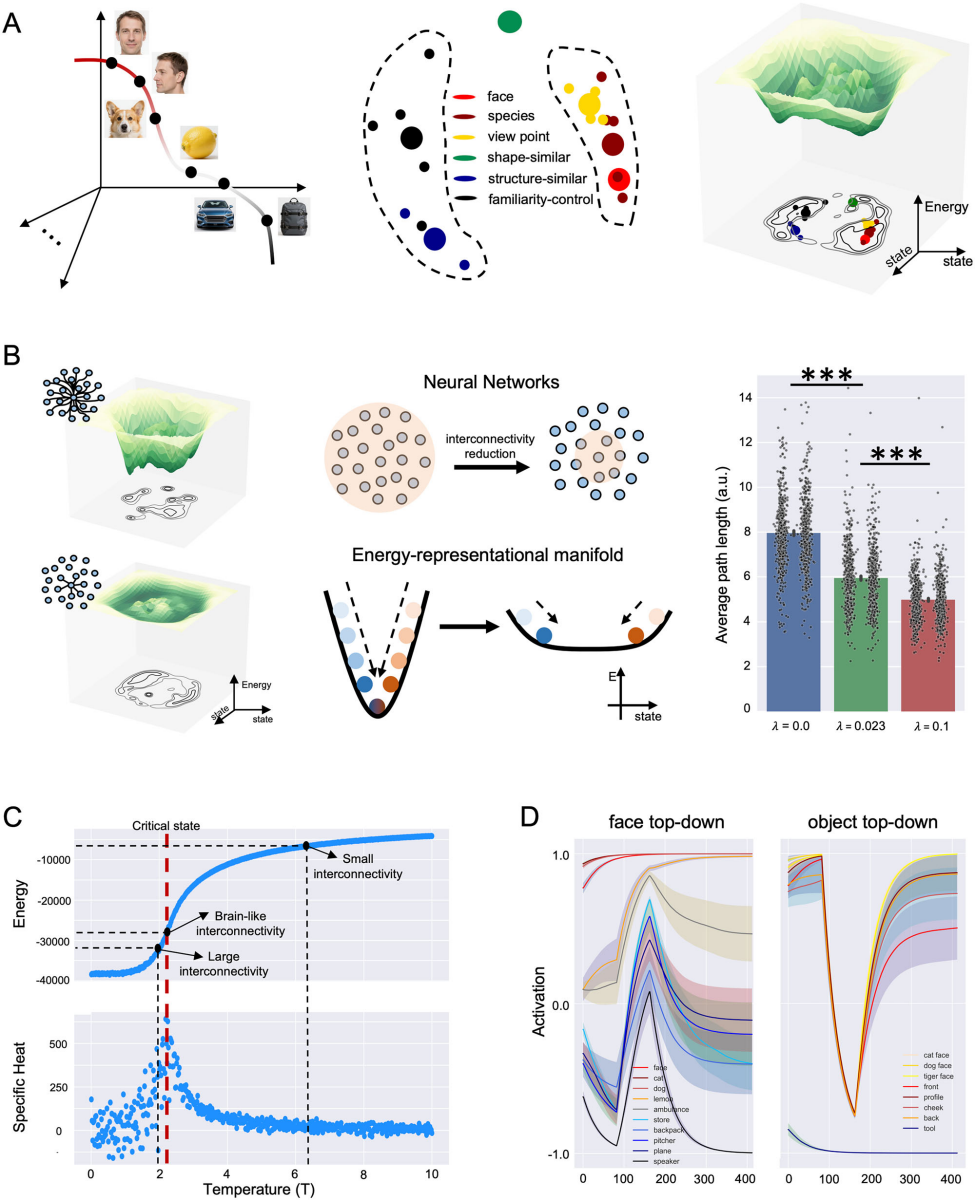
Energy-representational manifold and its characteristics. **A** Left: Schematic illustration of the object manifold in high-dimensional neural space. Face stimuli are located on the red line segment, while tool stimuli are positioned on the black line segment. Objects that share shapes or configurations with faces are situated in between. The images shown here were generated by artificial intelligence for illustration purpose only. Middle: The 2D representational manifold of object categories projected from the high-dimensional neural space via the parametric-UMAP method. Each small dot represents one object category at the basic level (e.g., cat face), and colors of the dots indicate object categories at the conceptual level (e.g., faces of different species). Big dots denote the center of object categories at the conceptual level. Right: Energy of neural states incorporated into the 2D representational manifold as a third axis, thus forming a 3D energy-representation manifold. Contour lines projected onto the representational manifold denote energy values, illustrating the shape and elevation of the energy. Contour lines close together indicate a steep slope, while lines spaced further apart indicate a gentler slope. **B** Left: Energy-representation manifolds constructed by models with a longer ($\lambda$ = 0.0, left) and shorter ($\lambda$ = 0.1, right) wiring length, respectively. Note that contour lines with a longer wiring length (top) are closer together than those with a shorter wiring length (bottom), indicating a steeper slope in the changes of energy in the manifold. Middle: Schematic illustration of the attractor regions in the energy-representation manifolds with different slopes. The steeper the slope, the longer the path that takes the initial neural state to travel to the stable state. Right: Average path lengths of attractor regions in models with different wiring lengths. Our model with the biologically inspired wiring length formed attractor regions that were neither too deep nor too shallow, suggesting a balanced dynamic. **C** Criticality. Hamiltonian value (top) and specific heat (bottom) at different temperatures of the Ising model. The Hamiltonian value for the model with $\lambda$ = 0.023 lay close to the critical point of the Ising model, whereas models with either a longer or a shorter wiring length resided in either order or chaos regions. **D** Cognitive impenetrability. Left: A top-down signal of faces was applied to all neurons (indicative of the presence of faces), while inputs were continuously presented objects (e.g., lemon, ambulance, and backpack). Right: A top-down signal of objects was applied to all neurons (indicative of the presence of objects), while input stimuli were continuously presented various faces. Lines denote time courses of activation averaged across all neurons in the face cluster along iteration steps, with shaded areas denoting standard deviation. Time courses show the dominant effect of top-down signals (duration: from 80,000 to 160,000 steps); however, once the top-down signals were offset, the time courses resumed to their original trajectories, suggesting the cognitive impenetrability of the model. ***p < 0.001.

To investigate why, with this biologically inspired wiring length, our model achieved the optimal balance between specificity and generalization, we examined the energy distribution across the neural manifold as neural states evolved toward directions of lower energy during the stabilization of the network^71,73^. To do this, we developed a dimension reduction method, namely parametric Uniform Manifold Approximation and Projection (p-UMAP, see “Materials and methods”), which parametrizes the non-linear mapping from a high-dimensional space (Fig. 3A, left) to a 2D representational space (Fig. 3A, middle) using machine learning. Figure 3A (middle) shows the 2D representational space after the p-UMAP, where various types of faces were clustered together (i.e., generalization) and separated from non-face objects (i.e., specificity). Then, we calculated the network’s energy for each neural state to visualize the dynamics of the network. This energy is defined as:

1 $$H=-\frac{1}{2}{\sum }_{i,j}{w}_{{ij}}{s}_{i}{s}_{j}$$

where ${s}_{i}$ is the state of neuron $i$, and ${w}_{{ij}}$ is the connection weight from neuron $j$ to neuron $i$. Each network state corresponds to an energy value. These energy values were incorporated into the 2D representational space as a third axis, resulting in a 3D energy-representational manifold (Fig. 3A, right) (see “Materials and methods”). This manifold provides a comprehensive view of how neural states evolved, highlighting regions of stability (low energy) and instability (high energy), thereby offering insights into the network’s functional organization.

For comparison, we also generated energy-representation manifolds for networks with either a longer ($\lambda$ = 0.0) or a shorter ($\lambda$ = 0.1) wiring length (Fig. 3B, left). These wiring lengths were chosen to represent extremes in network connectivity, allowing us to observe the effects of short and long interconnectivity on neural dynamics. As expected, increased interconnectivity, resulting from a longer wiring length, yielded attractor regions characterized by a smaller radius and lower energy values (akin to a narrow but deep basin) (Fig. 3B, left). Furthermore, a smaller radius of attractor regions likely necessitated neural states to travel a relatively longer distance to settle at the basin of these attractor regions from their initial states. That is, to achieve a stable representation, the neural states may undergo extensive transformation (i.e., heavy representation compression) (Fig. 3B, middle). To quantify this intuition, we measured the path distances, the lengths of trajectories that neural states followed to reach the basin of attractor regions, for the networks with varying levels of interconnectivity, and confirmed this observation (one-way ANOVA *F*(2,2997) = 1137.31, *p* < 0.001; *ps* < 0.001, Tukey HSD) (Fig. 3B, right).

Through this heavy compression, only the most critical features of stimuli were preserved, facilitating the achievement of higher generalization. In contrast, the network with lower interconnectivity minimally transformed the representation of a stimulus by maintaining a high-dimensional space. This minimal compression allowed the stimulus to retain its unique properties crucial for achieving higher specificity. Further formal proof (for details, see Appendix 1 in the Supplementary Information) confirms this visual observation that increased interconnectivity greatly compresses the representation, reducing dimensionality and enhancing generalization, while decreased interconnectivity maintains higher dimensionality, enhancing specificity.

The energy distribution in the neural manifold indicates that our model, with the wiring length from the human cortex, formed attractor regions that were neither too deep nor too shallow, suggesting a balanced dynamic. Accordingly, an interesting question arises regarding the characteristics of the neural network with this type of structured interconnectivity. One characteristic is stability versus adaptability of neural states in the face of perturbations arising either intrinsically (e.g., spontaneous neuronal firing) or extrinsically (e.g., top-down modulation). Neural states are less stable when neural manifolds are marked by shallow attractor regions (i.e., high energy); thus, neural states are prone to transitioning to nearby attractor regions upon encountering perturbations, indicating a propensity towards high adaptability but low stability^77,78^. In such a case, although these networks possess high specificity, their performance is easily influenced by perturbations, which result in chaotic behaviors. In contrast, when neural manifolds are marked by deep attractor regions (i.e., low energy), neural networks showcase resilience to both intrinsic and extrinsic perturbations^77,78^. However, these networks lose their adaptability, making it difficult to dynamically adjust to different task demands, resulting in ordered (stereotypical) behaviors. In our model, the attractor regions shaped by the biologically inspired wiring length were neither too deep nor too shallow, suggesting that the network likely operated near a critical state, a dynamic regime situated between order and chaos. In neural systems, operating close to this critical boundary is theorized to confer substantial computational advantages, such as optimized information processing, maximal sensitivity to external inputs, and efficient adaptability to new conditions, all while maintaining robustness to noise and perturbations^79–81^. Previous empirical and computational studies using models such as the Ising model have suggested that resting-state neural activity often resides close to this critical point^82–84^. Here, we investigated whether our model also exhibits dynamics consistent with criticality.

To do this, we calculated the Hamiltonian value and specific heat at each temperature, using an Ising model with the same size of the network, and identified the critical temperature corresponding to the peak of the specific heat (Fig. 3C). In parallel, we used noise as input to simulate the resting-state neural activity of the network (see “Materials and methods”), and calculated its Ising Hamiltonian value in comparison with that of the Ising model’s critical state. We found that the Hamiltonian value at the network’s resting state approximately coincided with the critical state of the Ising model (Hamiltonian value: −29,476; critical state: −28,897) (Fig. 3C). In contrast, neural networks with either a longer (Hamiltonian value: −31,433) or a shorter (Hamiltonian value: −6701) wiring length resided in either ordered or chaotic regions, respectively, suggesting that deviations from this optimal wiring length propel the network away from the critical state. Therefore, with its biologically inspired wiring length, our model’s resting‑state Hamiltonian closely matched the critical value predicted by the Ising model, suggesting that the model’s dynamics were consistent with operation near criticality.

To explore how the model’s near-critical dynamics influence face recognition performance, we applied a simplified, idealized top‑down perturbation during ongoing bottom‑up stimulus processing to examine the model’s cognitive impenetrability of perceptual representations^85^. Cognitive impenetrability is a signature of perceptual modularity characterized by its resilience to modulation by higher-level cognitive processes such as expectations, prior knowledge, or attention states. Accordingly, certain processes in perceptual modules remain relatively insulated, or encapsulated, from cognitive influence. For instance, recognizing a familiar face or identifying basic visual features typically remains stable even when cognitive context or expectation is altered. Evaluating cognitive impenetrability thus allows us to test whether the model’s perceptual representations remain robust despite competing top-down influences, providing evidence for genuine modularity at the level of neural population dynamics.

Specifically, two types of top-down perturbations were examined: (1) a top-down signal of faces applied in the presence of non-face objects (false alarm condition), simulating scenarios where non-face objects are incorrectly identified as faces, and (2) a top-down signal of non-face objects applied when faces were present (miss condition), representing the failure of recognizing actual faces due to interference from non-face signals. We asked whether our model was sensitive to these top-down signals and, more importantly, whether it resumed its inherent specificity and generalization after the withdrawal of these top-down signals.

For simplicity, top-down signals were exerted directly on the neurons in the model by an additional value of 2 to the neurons in the face cluster and −2 to the rest neurons to denote the presence of faces in the false alarm condition. In the miss condition, the values of the neurons in the object cluster were increased with an additional value of 2, and those of the rest neurons, including neurons in the face cluster, decreased with an additional value of −2 to denote the presence of non-face objects. In this way, the top-down signals intentionally guided the model towards a specific state (faces or objects). We then tracked the dynamics of neural activation before, during, and after these top-down signals to observe our model’s response to perturbations. In the false alarm condition, the activation of face-responsive units was first enhanced due to the top-down face signal, even in the presence of non-face objects. However, following the withdrawal of the top-down face signal, the model’s state reverted to its original dynamics, with a significant decrease in neurons’ responses to non-face objects (Fig. 3D, left), indicating that our model was able to recover its original specificity once the perturbation was removed. In contrast, in the miss condition, the neurons’ responses to faces were suppressed due to the top-down object signal. However, the model’s state resumed its dynamics upon signal removal (Fig. 3D, right), illustrating its ability to restore its generalization after the perturbation. Therefore, while activation patterns within the face‑selective cluster initially shifted toward the imposed top-down perturbation, they returned promptly to their original, stimulus‑driven trajectory once the perturbation was removed, suggesting the model’s robustness in representing faces against this simplified, idealized perturbation.

## Discussion

In this study, we address the challenge of balancing specificity and generalization in object recognition through a population coding perspective, proposing that the dimensionality of the neural space is crucial for achieving this balance. By examining macaques’ IT cortex, particularly the TEO and TEa, and employing computational modeling, featuring intra-module excitatory and inter-module inhibitory connections, we establish a causal link among abstraction, interconnectivity, and dimensionality. Specifically, we demonstrate that proper interconnectivity among neurons created an optimal dimensionality of the neural space, allowing for efficient energy distribution across the representational manifold embedded in the neural space to balance specificity and generalization. A key aspect of interconnectivity is the wiring length derived from biological brains, pivotal in achieving this optimal balance by ensuring efficient connectivity within the network. Additionally, this wiring length facilitates the network’s maintenance in a criticality-like state and promotes the formation of cognitive modules. In conclusion, our study underscores the role of efficient neural wiring in balancing detailed and generalized representations, providing an example of the principle in life science that structure determines functionality.

In terms of dimensionality of the neural space, specificity and generalization on object recognition can be achieved simultaneously in high-level visual cortex. In fact, in macaques’ IT cortex, the TEO and TEa achieved both specificity and generalization, and only differed quantitatively in their preference, with the TEO inclined to specificity and the TEa to generalization. This finding, observed at the population level, complements rather than contradicts hierarchical computational models of object recognition, operating at the neuronal level, where specificity is achieved through neurons tuned for simple features, and generalization is achieved through the pooling operations that combine information from multiple neurons through interconnectivity^7,8,86,87^. Indeed, this hierarchical structure may be facilitated by interconnectivity, allowing neurons with varying sensitivities to form complex networks where specificity and generalization are achieved simultaneously. On one hand, interconnectivity among neurons can sharpen their tuning curves by refining responsiveness to specific stimuli and reducing reactions to irrelevant ones through mechanisms such as lateral inhibition^88,89^, recurrent excitation^90,91^, and synaptic weight adjustment^92,93^. Additionally, interconnectivity can foster non-linear mixed selectivity by enabling neurons to integrate inputs from diverse sources and respond to combinations of features rather than individual elements^25,35,40,94,95^. Neurons with either narrower tuning curves^22,25,96–98^ or non-linear mixed selectivity^94,95,99,100^ increase the dimensionality of the neural space, enhancing networks’ ability to encode complex and diverse stimuli to achieve specificity in object recognition. On the other hand, interconnectivity can also reduce the dimensionality of the neural space by clustering neurons with similar response properties through Hebbian plasticity^101,102^, pooling and integrating the inputs to compress the input space^103,104^, enabling functional segregation to allow different groups of interconnected neurons to specialize in processing specific types of information^105^ and recurrent inhibition to suppress irrelevant or redundant responses^106,107^. These mechanisms create efficient, abstract, and generalizable representations of objects, thereby reducing dimensionality to generalize across different instances of the same object category. Notably, neural populations have finite representational capacity, and therefore only category distinctions aligned with dominant, behaviorally informative axes can be reliably encoded. For example, our selection of the spiky versus stubby distinction reflects this principle, following known organizing axes in IT cortex^39^. Thus, structured interconnectivity may implement dimensional compression, selectively compressing variance along behaviorally relevant axes to promote within‑category generalization, while preserving variance in orthogonal dimensions to maintain between‑category specificity. In summary, interconnectivity crucially modifies neurons’ response profiles, and thus dynamically regulates activation patterns of the network and its dimensionality, ensuring the network can efficiently process complex information and achieving both specificity and generalization essential for robust object recognition.

To satisfy these multiple functionalities, interconnectivity among neurons needs to be structured. Indeed, in our baseline RNN (RNN_h_, a fully connected Hopfield-like network with uniform global recurrence), uniform connectivity impaired the formation of distinct neural representations and hierarchical structures critical for fine-grained object recognition. Thus, the RNN_h_ possesses only generalization but lacks specificity. In our model, we adopted Hebbian and anti-Hebbian rules to form structured interconnectivity. Hebbian rule reinforces the desired patterns, while anti-Hebbian rule ensures that these patterns remain distinct and non-overlapping, resulting in distinct attractors corresponding to low-energy, stable configurations^70,102^. Further, the analysis on the energy-representation manifold illustrates that varying levels of interconnectivity shape the attractor regions’ geometry, with higher interconnectivity resulting in narrower, deeper attractor regions that favor generalization and lower interconnectivity creating broader, shallower attractor regions that enhance specificity. The levels of interconnectivity are influenced by wiring lengths, with a longer wiring length favoring a higher level of generalization. This finding is consistent with the hypothesis based on fMRI studies that the level of abstraction is linked to the spatial scale of representations^2,108^, where more abstract representations are implemented in larger spatial scales. Specifically, the wiring length derived from the biological brain resulted in attractor regions in the energy-representation manifold that best balance specificity and generalization. Interestingly, this particular wiring length places the model into a dynamical regime approaching the near-critical state. Such near criticality is thought to optimally balance sensitivity to perturbations for adaptability and noise immunity for stability. Under these balanced dynamics, a face-selective cognitive module likely emerges, exhibiting a key property analogous to cognitive impenetrability. This modular characteristic enables the network to preserve perceptual representations despite external cognitive influences. Future studies should rigorously quantify criticality using established statistical markers (e.g., long-range correlations and avalanche statistics) and assess the robustness of modularity under biologically realistic top-down manipulations (e.g., attention and prior knowledge). Such investigations would more definitively test the hypothesis that structured interconnectivity gives rise to critical dynamics and perceptual modularity.

In summary, our study explicitly focuses on anatomical interconnectivity, indexed empirically by dendritic spine density and represented in our computational model through the wiring length, to elucidate its causal impact on manifold geometry and the balance between representational specificity and generalization. Although this targeted approach provides clarity and interpretability, it introduces several noteworthy limitations. First, our computational model is static and does not incorporate mechanisms for online synaptic modification, limiting our ability to draw direct conclusions about experience-dependent neuroplasticity. Second, dendritic spine density in the IT cortex primarily indexes excitatory synaptic connections and therefore does not directly inform the organization or functional engagement of inhibitory circuits. Inhibitory mechanisms, such as lateral inhibition and competitive feedback^109–111^, substantially influence neuronal tuning and population-level dynamics. Thus, relying primarily on spine density introduces an inferential gap between our empirical data and the model’s assumed balance between excitation and inhibition arising from the Hebbian and anti‑Hebbian learning rules. Third, additional architectural determinants that could affect representational properties, including recurrent kernel characteristics, network depth, and non-linear activation functions, were not explored in this study. To overcome these limitations, future research should integrate online synaptic plasticity mechanisms within the structured interconnectivity framework, explicitly incorporate inhibitory circuit constraints, and systematically assess the effects of a broader array of architectural variables. Such investigations would allow researchers to quantify how these factors influence manifold dimensionality, category separability, and overall representational geometry at the neural population level. Furthermore, longitudinal empirical studies examining changes in IT cortical connectivity and representational manifolds before and after learning could directly test the hypothesis that structured interconnectivity dynamically modulates the specificity-generalization balance through experience-dependent plasticity.

## Materials and methods

### Stimuli

#### Stimuli for single-unit recordings

The stimuli used for single-unit recordings in macaques’ IT cortex included 51 exemplars from six different categories: animals, vehicles, faces, vegetables, houses, and tools. Each stimulus was presented in 24 different views to capture a range of visual perspectives. The face stimuli were generated by FaceGen (https://www.facegen.com) with randomly selected parameters to ensure variability. Stimuli for other categories were sourced from a 3D model repository (https://www.3d66.com). The 24 views for each exemplar were created using 3DMAX software. For additional details on the stimulus design, see Bao et al.^39^.

#### Stimuli for the model

To examine face specificity, we primarily used stimuli from the ImageNet1000(mini) dataset^112^, which contains 1000 categories with approximately 40 exemplars per category. Specifically, we selected animal faces from the cat and dog categories. Lemons were included because they mimic the external feature of faces (i.e., roundness), while vehicles, specifically ambulances and airplanes, were chosen due to their face-like configurations. To ensure diversity among non-face object categories, we randomly selected four categories: two from outdoor scenes (stores and backpacks) and two from indoor scenes (pitchers and speakers). Face stimuli were selected from the LFW Face Database^113^, as the face category was not included in the ImageNet1000(mini) dataset. In total, ten categories were used for the face specificity test (for stimulus examples, see Fig. S7).

To examine face generalization, we included faces from different species (cat, dog, and tiger), randomly selected from the ImageNet1000(mini) dataset, with 10 exemplars per category. Additionally, we utilized FaceGen to generate 15 human faces from the frontal view (0°) and their corresponding profile (90°), cheek (faces rotating 135°), and back (180°) views. Importantly, the human faces used in the face generalization test were different from those used in the face specificity test, as well as from the natural stimuli used to train AlexNet, to rigorously assess the model’s generalizability. Three types of tools (vacuum cleaners, electric drills, and knives) from the HCP dataset^61^ were also included as baseline objects. In total, ten categories (3 animal faces, 4 views of human faces, and 3 tools) were used in this test (for stimulus examples, see Fig. S7).

### Analyses on single-unit data

#### Neuron selection

We analyzed single-unit data from Bao et al.^39^, which were originally acquired from the IT cortex of two head-fixed male macaques as they passively viewed the stimuli. The stimuli were presented on a CRT monitor in a random order, with a screen view angle of 27.7° × 36.9° and a stimulus view angle of 5.7°. The fixation point had a diameter of 0.2°. The position of the macaques’ eyes was monitored using an infrared eye-tracking system (ISCAN) to ensure accurate fixation during stimulus presentation. Electrical signals were recorded from a total of 483 neurons in the IT cortex, with 106, 175, and 201 neurons from the TEO, TEp, and TEa, respectively. Of those, 10, 190, 67, and 216 neurons were located in face-, body-, stubby-, and spiky-responsive patches, respectively. For additional details on the recordings and the characteristics of these neurons, see Bao et al.^39^.

To select neurons for the analysis of specificity and generalization, we applied two specific criteria: (1) The average response of a neuron to a category had to exceed twice the maximum of the average response to other categories, ensuring that the neuron showed a strong preference for that category. (2) The number of neurons satisfying the first criterion had to be greater than five, which is the minimum required to support reliable populational coding. As a result of these criteria, only neurons responsive to the animal category met the inclusion requirements. The number of neurons responsive to other categories did not exceed five, and therefore, they were excluded from further analyses. For detailed information on the number of category-responsive neurons, see Table S1.

A similar procedure was used to select spiky-responsive neurons. Spikyness was defined as a stimulus feature characterized by protrusion, with stimuli possessing this feature labeled as spiky stimuli (for details, see Bao et al.^39^). Of the total 51 exemplars, the spiky stimuli contained 11 exemplars, while the non-spiky (stubby) stimuli contained 9 exemplars. Each exemplar was presented in 24 different views. A neuron was defined as spiky-responsive if its response magnitude to spiky stimuli was at least twice as large as its response to non-spiky stimuli. In the replication analysis conducted on each macaque, spiky-responsive neurons were extracted from each macaque separately, following the same procedure.

#### Assessment of specificity and generalization

To quantify the specificity of animal-responsive neurons, the specificity score was defined as the ratio of the average response magnitude of the animal-responsive neurons to animal stimuli compared to the maximum average response magnitude to non-animal stimuli. A higher specificity score indicates greater specificity for animal stimuli. To ensure the robustness of this measure, we randomly selected exemplars from each category 20 times, calculating a specificity score for each iteration. By averaging these 20 specificity scores, we obtained a final specificity score for each animal-responsive neuron.

To quantify the generalization of animal-responsive neurons, we calculated two types of generalization scores. To measure generalization across different animal exemplars (e.g., dogs, cats, birds, ducks, horses, and fish), the generalization score was defined as the ratio of the minimum to maximum response magnitude of an animal-responsive neuron in response to all exemplars. A higher score indicates that the neuron responds consistently across different animal exemplars, suggesting greater generalization. To measure generalization across different views of the same exemplar, the generalization score was defined as the ratio of the minimum to maximum response magnitude across different views of the same exemplar. A higher score indicates greater view invariance, meaning the neuron responds consistently to different views of the same exemplar. The calculation for both scores was repeated 20 times to obtain a reliable generalization score for each animal-responsive neuron. Additionally, we used standard deviations to measure generalization, as the smaller the standard deviation of neural responses to different exemplars of the same category (or different views of the same exemplar), the higher the generalization.

The specificity and generalization of the spiky-responsive neuron were assessed using the same procedure.

We also employed the representational similarity analysis (RSA) to measure specificity and generalization at the population level. For specificity, we calculated Pearson correlations between the average representation of animal stimuli for animal-responsive neurons and each non-animal stimulus category. The average of these correlation values yielded the specificity score. Lower score indicates stronger specificity of the group neuron to animal category. To measure generalization across different animal exemplars, the representations of animal-responsive neurons to animal examples were extracted, and representational similarity matrix (RSM) was calculated by computing pairwise Pearson correlations. The upper triangular portion was averaged to yield the generalization score. Higher score indicates more consistent responses to different animal examples, signifying stronger generalization ability. To measure generalization across different views, the score was defined as the representation of animal-responsive neurons for different animal views. The upper triangular portion of the RSM was averaged to yield the generalization score. Higher score indicates stronger invariance to views. To ensure robustness, each metric is computed by randomly selected samples, calculating scores, and averaging the results over 20 iterations.

#### Effective dimensions and radii of point clouds

Chung et al.^19^ developed a theory of linear divisibility of manifolds, which defines the effective dimension and radius of a single manifold using anchors at the edges of the manifold. Specifically, the effective dimension is defined as the spread of these anchor points along the different axes of the manifold, while the radius is the total variance of the anchor points normalized by the mean distance from the center of the manifold. To apply Chung et al.’s method to our data, we first randomly selected 100 neurons in each of the three regions (TEO, TEp, and TEa). We then used the Louvain algorithm, a method for detecting communities by optimizing a quality function known as clustering^114^, to segment all neural representations into several representational manifolds. The Louvain algorithm works by iteratively moving nodes between communities to maximize clustering, resulting in cohesive manifold groups. Finally, we applied Chung et al.’s method to calculate the effective dimension and radius of each manifold. This procedure was repeated 20 times to obtain reliable values for the TEO, TEp, and TEa.

For the analyses performed on each macaque, the same procedure was followed, except that only 30 neurons were randomly selected for each region due to the smaller number of neurons available. The calculation was repeated 30 times to ensure robustness in the results for each macaque.

### Brain-inspired computational model

The computational model consists of two main components. (1) Image encoder. The first component is an image encoder, implemented using a deep convolution neural network (AlexNet), which transforms images into vector representations (i.e., object space) to serve as input for the second component of the model. (2) Simulation of human visual temporal cortex. The second component simulates the human VTC using a stochastic Hopfield-like neural network with lateral connections. Critically, the wiring length of the lateral connections is derived from the estimation of the human brain. Together, these two components enable the computational model to process images in a manner that reflects the structure and function of the human visual system.

#### Recurrent stochastic Hopfield neural network

In our previous study, we demonstrated that a hybrid model consisting of a pre-trained AlexNet and a self-organizing map (SOM) could preserve the topological organization of fine-scale functional regions in the human VTC^59^. However, neurons in the human VTC are characterized by extensive lateral connections, particularly among neighboring neurons. These lateral connections, which can be either excitatory or inhibitory, contribute to the dynamical properties of the network, enabling complex processing and integration of visual information. Building on our previous work, in this study, we implemented lateral connections in the SOM to better simulate the functionality of the human VTC, with a particular focus on the fusiform face area (FFA), a region specialized for face processing. Importantly, by incorporating these lateral connections, our model enables a direct exploration of the causal link between structured interconnectivity, the dimensionality of neural space, and the balance between specificity and generalization in object recognition.

We incorporated excitatory and inhibitory connections between neurons in the SOM, and the weights of these connections were trained using 4 memory patterns. These memory patterns represent the activation patterns corresponding to faces, daily objects, places, and bodies in our original hybrid model. In accordance with classical Hopfield neural networks, each of these patterns was binarized, assigning a value of +1 to neurons in the functionally activated areas and −1 to neurons that were not activated. This binarization process ensures that the network can store and retrieve these patterns, reflecting the distinct functional areas observed in the human brain. We use ${x}_{i}^{\mu }$ to denote the activation state of the *i*th neuron at the *μ*th pattern:

$${x}_{i}^{\mu }=\left\{\begin{array}{c}+1,{when\; neuron}i{is\; activated}\,\\ -1,{when\; neuron}i{is\; not\; activated}\end{array}\right.$$

These four memory patterns serve as functional areas within the SOM, guiding the organization of neurons in the network. Lateral connections between neurons were established based on Hebbian and anti-Hebbian learning rules, which allow the network to strengthen or weaken connections depending on the correlated activity of the neurons. These rules transformed the original SOM into a recurrent neural network, enabling dynamic signal transmission across the network. Specifically, the weights between neuron *i* and *j* are calculated as a weighted sum:

$${w}_{{ij}}=\frac{{\gamma }_{{ij}}}{N}\mathop{\sum }\limits_{\mu }^{4}{x}_{i}^{\mu }{x}_{j}^{\mu }\,\left(\mu =1,2,3,4\right)$$

$${\gamma }_{{ij}}=\left\{\begin{array}{c}\,1,\,w.p.{\,e}^{-\lambda {d}_{{ij}}}\\ 0,{otherwise}\end{array}\right.$$

whereas the factor ${\gamma }_{{ij}}$ determines whether a connection exists between neuron *i* and *j*, with ${\gamma }_{{ij}}$ equal to 1 with probability ${e}^{-\lambda {d}_{{ij}}}$ or 0 otherwise. Here, ${d}_{{ij}}$ represents the Euclidean distance between neuron *i* and *j*. As the distance ${d}_{{ij}}$ increases, the probability of a connection decreases, making ${\gamma }_{{ij}}$ and the corresponding weight ${w}_{{ij}}$ more likely to be 0. The parameter $\lambda$ is a constant that determines the range of lateral connections, controlling how rapidly the probability of a connection decreases with distance and thus effectively limiting the influence of neurons on each other based on their spatial separation within the network. The value of $\lambda$ was derived from estimation of the human brain (i.e., a biologically constrained parameter)^65^.

In our model, we set the parameter $\lambda$ to represent the wiring length of lateral connections in the human temporal lobe, and kept it constant throughout the model training. Though the exact value of $\lambda$ in the human temporal lobe has not been directly measured, a recent study by Theodoni et al.^65^ estimated its value to be approximately 0.1, based on comparative studies of regions homologous to the human temporal lobe across different species. To fine-tune the parameter $\lambda$ for our model, we aligned the human VTC with the lattice of the model. Specifically, we first calculated the half-height width of the function ${{e}}^{-\lambda {d}_{{ij}}}$ as 6.93 mm when $\lambda$ is 0.1. Next, we computed a geodesic distance map (GDM) with a geodesic distance of 6.93 mm around each vertex of the human VTC. For both the left and right human VTC, we mapped the GDM of each vertex onto the lattice using a deformation field, and then calculated the average number of neurons in the lattice that the GDM of one vertex occupied. As a result, when the parameter $\lambda$ in the model was set to 0.023, the number of neurons occupied by the half height and width of ${{e}}^{-\lambda {d}_{{ij}}}$ in the model approximately matched the average number of neurons occupied by the GDM of one vertex in the human VTC. Therefore, the parameter $\lambda$ in our model was set to 0.023, closely approximating the wiring length of lateral connections in the human temporal lobe.

#### Network dynamics

Dynamics are also implemented in our model to update the neural states of neurons, simulating the dynamic behavior of biological neural systems. In accordance with classical stochastic Hopfield neural networks, the state of each neuron, which can be either +1 (activation) or −1 (deactivation), is influenced by neurons to which it is connected during each iteration. That is, at each iteration, neuron *i* is randomly selected from neurons in the lattice. This neuron then updates its state ${s}_{i}$ by integrating its current activation state with an input signal ${b}_{i}$:

$${b}_{i}=\sum {w}_{{ij}}({s}_{j}+{{{\mathcal{H}}}}_{j})$$

where ${b}_{i}$ is a weighted sum of the state ${s}_{j}$ of neuron *j* and an external input ${{{\mathcal{H}}}}_{j}$, which represents a signal from outside the VTC that influences neuron *j*. The state ${s}_{i}$ of this neuron becomes +1 (activation) with a conditional probability $p({s}_{i}=+1|{b}_{i})$, given by:

$$p({s}_{i}=+1|{b}_{i})=\frac{1}{1+{e}^{-\beta {b}_{i}}}$$

This probability is determined by a sigmoid function, where the input signal ${b}_{i}$ is weighted by the parameter $\beta$. The magnitude of $\beta$ controls the steepness of the sigmoid function, capturing the intrinsic randomness in the model’s dynamics. A larger $\beta$ results in a steeper sigmoid function, meaning the system is less influenced by noise and more deterministic in its response. In our model, $\beta$ was set to 100 to simulate a very low-level noise, ensuring that the model behaves in a highly deterministic manner. As a result, the model functions as an attractor network, capable of stabilizing around the four memory patterns corresponding to faces, bodies, places, and daily objects.

### Invariance and corresponding neural geometry

#### Specificity and generalization

To assess the specificity and generalization of the face cluster in our model, we averaged the neural states of all neurons within the cluster once the network was stabilized. Specifically, in each trial, an image from a category was processed by the model, resulting in an initial state for each neuron. The neural states were then updated over 150,000 iterations in the presence of the input image until a stable state was achieved. Our preliminary study indicated that 150,000 iterations were sufficient for the model to stabilize. To depict the developmental trajectory of the neural states, during each iteration, we calculated the mean activation magnitude of the neurons within the face cluster. This procedure was applied to both specificity and generalization tests, using different image sets (see “Materials and methods”). The specificity and generalization scores were calculated using the same methods as those applied to the neurophysiological data (see “Materials and methods”). To compute the balance score at each interconnectivity level, we first normalized the specificity and generalization scores separately to a 0–1 range using min–max scaling. These normalized values were then summed, yielding a balance score for each level, with higher scores indicating a more optimal balance between specificity and generalization.

For comparison, we also measured the specificity and generalization scores of a forward neural network (AlexNet, FNN_a_) and a recurrent neural network (Hopfield Neural Network, RNN_h_). Since the FNN_a_ does not inherently possess face clusters, here we decoded face responses by connecting a small neural network as a decoder to the FNN_a_^115^. This decoder consists of two layers. The first layer contains 1000 units with a ReLU activation function, which receives a 1000-dimensional vector output from the FNN_a_ and reduces it to a 100-dimensional vector for input to the second layer. The second layer contains 100 units and outputs 2-dimensional vector, which was used for the binarized classification of faces and non-face objects. The unit in the second layer corresponding to the classification of faces was referred to as the face unit. To train the decoder, we used natural face stimuli from the LFW face dataset^116^, which contains a total of 5749 face images, and natural object stimuli from the Caltech256 dataset^117^. To ensure that the object stimuli contained no faces and to match the number of the face stimuli, we used an in-house face-detection toolbox based on VGG-Face^118^ to remove images containing faces from the Caltech256 dataset, ultimately selecting 5749 non-face object images. The decoder was trained using the stochastic gradient descent optimizer^119^. Note that all parameters of the FNN_a_ were frozen during the decoder training. Once the decoder training was complete, we evaluated the specificity and generalization scores with the activation magnitudes of the face unit in the second layer of the decoder in response to faces from the specificity and generalization dataset (see “Materials and methods”). Finally, we calculated the specificity and generalization scores using the same methods as those applied to our model.

We followed the method provided by Tang et al.^120^ to construct the RNN_h_. Specifically, recurrent connections were implemented in the last layer of AlexNet (fc3 layer) to form a Hopfield network, referred to as RNN_h_. These recurrent connections were trained using images from the HCP dataset, following the Hebbian learning rule (for detailed training methods, see Tang et al.^120^). Once the training was complete, we decoded the RNN_h_ using images from the specificity and generalization dataset (see “Materials and methods”). To obtain face-responsive and object-responsive units, we employed a support vector machine (SVM) to perform binary classification of faces and objects (for detailed analysis methods, see Tang et al.^120^). After identifying the face-responsive units, we used the same methods as those applied to our model to calculate the specificity and generalization scores.

#### Energy-representation manifold

We used the same methods as those applied to the neurophysiological data to obtain the effective dimensions and radii of the representational manifold formed by 1000 neurons randomly selected from the entire neuron population. To visualize the manifold in a 2D surface, we enhanced the traditional UMAP method by introducing a parametric approach, which utilizes a forward neural network to parametrize the non-linear mapping between the high-dimensional data space and the two-dimensional space for visualization. This approach, referred to as parametric-UMAP, offers an advantage over traditional UMAP or other manifold learning methods by enabling the learning of non-linear mappings directly from the data. Specifically, we first generated a high-dimensional neural manifold using the model with 1000 randomly selected natural images from the ImageNet validation set^112^. We then applied the UMAP to project this manifold onto a 2D surface. In this process, a feedforward neural network was used to learn the non-linear mapping from the high-dimensional space to a two-dimensional space, resulting in the 2D coordinates that represent the geometry of the high-dimensional manifold. This feedforward neural network consists of three layers, with the first, second, and third layer containing 1000, 100, and 2 units, utilizing the ReLU activation function, respectively.

Since our model is a Hopfield-like neural network, the states of the model evolve towards states with smaller energy, reflecting the model’s tendency to minimize its energy. To depict this dynamic characteristic, we calculated the energy for each state of the network from the initial state to the stable state using the following equation:

$$H=-\frac{1}{2}\mathop{\sum }\limits_{i,j}{w}_{{ij}}{s}_{i}{s}_{j}$$

where *H* denotes the energy as a function of the network’ state, ${s}_{i}$ and ${s}_{j}$ are the states of neuron $i$ and *j*, respectively, and ${w}_{{ij}}$ is the connection weight from neuron $j$ to $i$. Since each network state corresponds to a specific energy value, we incorporated these energy value as a third axis alongside the 2D representational space obtained from the parametric-UMAP, creating what we refer to as the energy-representation space. This 3D space encompasses the representational manifold and its corresponding energy values. However, traversing the entire representation space to produce a complete energy-representation manifold is impractical due to the vast number of possible states and energy values (${2}^{40,000}$, as the lattice size of 200 × 200). To address this, we generated a proxy energy-representation manifold by performing three-dimensional interpolation, combining the energy values from 1000 representations obtained using the parametric-UMAP method.

To calculate the path distance that neural states followed from their initial states to reach the basin of attractor regions, we recorded one representation every 10,000 iterations during a total of 150,000 iterations, resulting in 15 representations for each stimulus. We then computed the average Euclidean distance between each pair of neighboring representations within the energy-representation manifold. These distances were integrated to determine the total path distance for each stimulus. Finally, we averaged the path distances of all 1000 stimuli used to form the manifold, providing a single measure of the path distance for the entire energy-representation manifold. This distance captures the overall trajectory of neural states as they converge towards attractor regions, reflecting the dynamics of the model.

### Criticality and cognitive impenetrability

#### Criticality estimated by Ising model

We used the Ising model to explore the criticality of our model, which is a fundamental model in statistical mechanics that describes a spin lattice system^121^. Each spin can be in one of two states: up or down. The model considers the interactions between neighboring spins that are first-ring adjacent, meaning they are directly connected. The Hamiltonian *H* of the Ising model represents the total energy of the system and is defined as:

$$H=-\,J\mathop{\sum }\limits_{ < i,j > }{s}_{i}{s}_{j}$$

where *J* is a constant representing the interaction strength between neighboring spins (typically set to 1), ${s}_{i}$ and ${s}_{j}$ denote the spin states of site *i* and *j*, respectively. The summation is carried out over all pairs of adjacent spins, reflecting the interaction that contribute to the system’s energy.

The Ising model was set to have the same size as our model (i.e., 200 × 200) to allow for a direct comparison between the two systems. To calculate the Hamiltonian values of the Ising model, we employed Monte Carlo simulations over a range of 500 isometric temperatures, spanning from 0.001 to 10^122^. At each temperature, the Monte Carlo simulations were run for 16,000,000 iterations to allow the system to reach equilibrium. Following this equilibration period, an additional 1,600,000 sampling steps were performed to obtain the average Hamiltonian value. This extensive sampling ensures that the calculated Hamiltonian values are representative of the system’s equilibrium state. Finally, the Hamiltonian values were calculated for all 500 temperatures. The specific heat was determined as the partial derivative of the Hamiltonian with respect to temperature, which revealing how the energy of the system responds to changes in temperature.

To calculate the Hamiltonian value of our model, we simulated activation fluctuations during the resting state observed in the brain by introducing random noise inputs to our model. Specifically, each neuron of the model received a time-varying noise input of either −1 or +1. This procedure was used to simulate the bottom-up noise that the VTC receives from other cortical areas during the resting state. This process was run 300,000 steps, with the state of the model being recorded every 100 steps, thereby resulting in 3000 different resting states of the model. We then calculated the Hamiltonian values corresponding to each of these states and average them to obtain the overall average Hamiltonian value for the model during the simulated resting state.

#### Impenetrability of cognitive modules

We introduced top-down modulations to test the cognitive impenetrability of our model. To simulate the characteristics of top-down signals (e.g., a top-down signal of faces) observed in the brain that are briefer in time and stronger in magnitude than bottom-up signals^123–125^, an additional value of 2 was provided to the units in a cluster (e.g., the face cluster) and the rest neurons were provided with an additional value of −2, twice as strong as the predefined state of the model, and continuously presented bottom-up signals (i.e., the presence of stimuli throughout the simulation). Specifically, the bottom-up signals were presented for 80,000 iterations, followed by the simultaneous presence of both top-down and bottom-up signals for another 80,000 iterations. Finally, only the bottom-up signals were presented for an additional 250,000 iterations until the model reached the stable state. For every 1000 iterations, the average response magnitude of neurons in the face cluster was recorded. This data was used to depict the developmental trajectory of the model, showing how the model evolved under the influence of both top-down and bottom-up signals.

### Statistics and reproducibility

All quantitative analyses were carried out using custom code developed for this study (see “Data and code availability” for repository information). Unless otherwise stated, summary values are reported as mean ± standard error of the mean, and all statistical tests were two-tailed with a significance threshold of α = 0.05. The specific statistical test used (for example, one-sample or two-sample *t*-tests) and the corresponding test statistics and *p*-values are reported in the main text and figure panels.

For analyses based on single-unit recordings in macaque inferior temporal (IT) cortex, each data point corresponds to an independently recorded neuron. In these analyses, n denotes the number of visually responsive neurons included for a given cortical area (TEO, TEp, or TEa), pooled across the two monkeys unless otherwise indicated. When analyses were performed separately for each animal, n refers to the number of neurons recorded in that animal. Replicates for the biological data are therefore independent biological replicates, defined as different neurons recorded under the same experimental conditions. The total numbers of neurons and category- or feature-responsive subsets used in each analysis are provided in the main text and Supplementary Table S1.

For the brain-inspired network model, n refers to independent realizations of the model or independent runs of the full analysis pipeline. Replicates for the modeling results are independent technical replicates, defined as model instances generated with different random draws of input stimuli. Energy values were computed by obtaining from each individual network state across thousands of replicates. In contrast, group-level estimates for quantities such as effective dimensionality, manifold radii, specificity, and generalization indices were obtained by repeatedly calculating these indices on different random samples of neurons from the aggregate neural population response and averaging the results.

Several analyses involving random subsampling procedures (for example, the estimation of effective dimensionality and radii of point clouds across cortical areas, or the robustness checks of neuron-selection thresholds) were repeated multiple times with different random seeds. In these cases, the reported values correspond to averages over repeated runs (for example, 20 or 30 repetitions), and the associated variability reflects the dispersion across these independent repetitions. The robustness of the main findings to neuron-selection thresholds, subsampling schemes, and analysis choices is demonstrated in the supplementary figures (for example, Figs. S1–S6).

For analyses that are primarily descriptive (such as example single-neuron response profiles, schematic illustrations of the model architecture, or representative low-dimensional embedding of the energy-representation manifold), the same procedures were repeated across neurons, cortical areas and model instances using the datasets and pipelines described in the “Materials and methods” section; the figures show representative examples that are typical of the overall pattern observed.

### Reporting summary

Further information on research design is available in the Nature Portfolio Reporting Summary linked to this article.

## Supplementary information

Supplementary Information

Reporting summary

## Data Availability

The monkey single-cell data reported in this article are partially available at https://github.com/sdgds/Balanced-ITC.

## Code availability

All custom code has been deposited at https://github.com/sdgds/Balanced-ITC and is publicly available as of the date of publication.

## References

[1] Ullman, S. *High-level Vision: Object Recognition and Visual Cognition* (MIT Press, 2000).

[2] Grill-Spector, K. & Weiner, K. S. The functional architecture of the ventral temporal cortex and its role in categorization. *Nat. Rev. Neurosci.* **15**, 536–548 (2014). 24962370 10.1038/nrn3747 PMC4143420

[3] Zoccolan, D., Kouh, M., Poggio, T. & DiCarlo, J. J. Trade-off between object selectivity and tolerance in monkey inferotemporal cortex. *J. Neurosci.* **27**, 12292–12307 (2007). 17989294 10.1523/JNEUROSCI.1897-07.2007 PMC6673257

[4] DiCarlo, J. J., Zoccolan, D. & Rust, N. C. How does the brain solve visual object recognition?. *Neuron* **73**, 415–434 (2012). 22325196 10.1016/j.neuron.2012.01.010 PMC3306444

[5] Marr, D. *Vision: A Computational Investigation into the Human Representation and Processing of Visual Information* Vol. 2 (Henry Holt and Co. Inc., 1982).

[6] Felleman, D. J. & Van Essen, D. C. Distributed hierarchical processing in the primate cerebral cortex. *Cereb. Cortex* **1**, 1–47 (1991). 10.1093/cercor/1.1.1-a 1822724

[7] Riesenhuber, M. & Poggio, T. Hierarchical models of object recognition in cortex. *Nat. Neurosci.* **2**, 1019–1025 (1999). 10526343 10.1038/14819

[8] Rolls, E. & Deco, G. *Computational Neuroscience of Vision* (Oxford University Press, 2001).

[9] Riesenhuber, M. & Poggio, T. Neural mechanisms of object recognition. *Curr. Opin. Neurobiol.* **12**, 162–168 (2002). 12015232 10.1016/s0959-4388(02)00304-5

[10] Rust, N. C. & DiCarlo, J. J. Selectivity and tolerance (“invariance”) both increase as visual information propagates from cortical area V4 to IT. *J. Neurosci.* **30**, 12978–12995 (2010). 20881116 10.1523/JNEUROSCI.0179-10.2010 PMC2975390

[11] Yamins, D. L. & DiCarlo, J. J. Using goal-driven deep learning models to understand sensory cortex. *Nat. Neurosci.* **19**, 356–365 (2016). 26906502 10.1038/nn.4244

[12] Yamins, D. L. et al. Performance-optimized hierarchical models predict neural responses in higher visual cortex. *Proc. Natl. Acad. Sci. USA* **111**, 8619–8624 (2014). 24812127 10.1073/pnas.1403112111 PMC4060707

[13] Cadieu, C. F. et al. Deep neural networks rival the representation of primate IT cortex for core visual object recognition. *PLoS Comput. Biol.* **10**, e1003963 (2014). 25521294 10.1371/journal.pcbi.1003963 PMC4270441

[14] Khaligh-Razavi, S. M. & Kriegeskorte, N. Deep supervised, but not unsupervised, models may explain IT cortical representation. *PLoS Comput. Biol.* **10**, e1003915 (2014). 25375136 10.1371/journal.pcbi.1003915 PMC4222664

[15] Guclu, U. & van Gerven, M. A. Deep neural networks reveal a gradient in the complexity of neural representations across the ventral stream. *J. Neurosci.* **35**, 10005–10014 (2015). 26157000 10.1523/JNEUROSCI.5023-14.2015 PMC6605414

[16] LeCun, Y. & Bengio, Y. Convolutional networks for images, speech, and time series. in *The Handbook of Brain Theory and Neural Networks* (ed Arbib, M. A.) Vol. 3361, 1995 (The MIT Press, 1995).

[17] Kriegeskorte, N. et al. Matching categorical object representations in inferior temporal cortex of man and monkey. *Neuron* **60**, 1126–1141 (2008). 19109916 10.1016/j.neuron.2008.10.043 PMC3143574

[18] Chung, S. & Abbott, L. F. Neural population geometry: an approach for understanding biological and artificial neural networks. *Curr. Opin. Neurobiol.* **70**, 137–144 (2021). 34801787 10.1016/j.conb.2021.10.010 PMC10695674

[19] Chung, S., Lee, D. D. & Sompolinsky, H. Classification and geometry of general perceptual manifolds. *Phys. Rev. X* **8**, 031003 (2018).

[20] Mitchell-Heggs, R., Prado, S., Gava, G. P., Go, M. A. & Schultz, S. R. Neural manifold analysis of brain circuit dynamics in health and disease. *J. Comput. Neurosci.* **51**, 1–21 (2023). 36522604 10.1007/s10827-022-00839-3 PMC9840597

[21] Elmoznino, E. & Bonner, M. F. High-performing neural network models of visual cortex benefit from high latent dimensionality. *PLOS Comput. Biol.* **20**, e1011792 (2024). 38198504 10.1371/journal.pcbi.1011792 PMC10805290

[22] Cai, D., Liu, T. & Liu, J. Encoding of interdependent features of head direction and angular head velocity in navigation. *PNAS Nexus* **4**, pgaf320 (2025). 10.1093/pnasnexus/pgaf320 PMC12550893 41142968

[23] Jia, W., Sun, M., Lian, J. & Hou, S. Feature dimensionality reduction: a review. *Complex Intell. Syst.* **8**, 2663–2693 (2022).

[24] Cohen, U., Chung, S., Lee, D. D. & Sompolinsky, H. Separability and geometry of object manifolds in deep neural networks. *Nat. Commun.* **11**, 746 (2020). 32029727 10.1038/s41467-020-14578-5 PMC7005295

[25] Langdon, C., Genkin, M. & Engel, T. A. A unifying perspective on neural manifolds and circuits for cognition. *Nat. Rev. Neurosci.* **24**, 363–377 (2023). 37055616 10.1038/s41583-023-00693-x PMC11058347

[26] Yoon, I. H. et al. Tracking the topology of neural manifolds across populations. *Proc. Natl. Acad. Sci. USA* **121**, e2407997121 (2024). 39514312 10.1073/pnas.2407997121 PMC11573501

[27] Pezon, L., Schmutz, V. & Gerstner, W. Linking neural manifolds to circuit structure in recurrent networks. Preprint at *bioRxiv*10.1101/2024.02.28.582565 (2020). 10.1016/j.neuron.2025.12.047 41794030

[28] Mastrogiuseppe, F. & Ostojic, S. Linking connectivity, dynamics, and computations in low-rank recurrent neural networks. *Neuron* **99**, 609–623. e629 (2018). 30057201 10.1016/j.neuron.2018.07.003

[29] Langdon, C. & Engel, T. A. Latent circuit inference from heterogeneous neural responses during cognitive tasks. *Nat. Neurosci.* **28**, 665–675 (2025). 10.1038/s41593-025-01869-7 PMC11893458 39930096

[30] Dubreuil, A., Valente, A., Beiran, M., Mastrogiuseppe, F. & Ostojic, S. The role of population structure in computations through neural dynamics. *Nat. Neurosci.* **25**, 783–794 (2022). 35668174 10.1038/s41593-022-01088-4 PMC9284159

[31] Recanatesi, S., Ocker, G. K., Buice, M. A. & Shea-Brown, E. Dimensionality in recurrent spiking networks: Global trends in activity and local origins in connectivity. *PLoS Comput. Biol.* **15**, e1006446 (2019). 31299044 10.1371/journal.pcbi.1006446 PMC6655892

[32] Sporns, O. *Networks of the Brain* (MIT Press, 2016).

[33] Tsao, D. Y., Freiwald, W. A., Tootell, R. B. & Livingstone, M. S. A cortical region consisting entirely of face-selective cells. *Science* **311**, 670–674 (2006). 16456083 10.1126/science.1119983 PMC2678572

[34] Issa, E. B., Papanastassiou, A. M. & DiCarlo, J. J. Large-scale, high-resolution neurophysiological maps underlying FMRI of macaque temporal lobe. *J. Neurosci.* **33**, 15207–15219 (2013). 24048850 10.1523/JNEUROSCI.1248-13.2013 PMC3776064

[35] Barak, O., Rigotti, M. & Fusi, S. The sparseness of mixed selectivity neurons controls the generalization–discrimination trade-off. *J. Neurosci.* **33**, 3844–3856 (2013). 23447596 10.1523/JNEUROSCI.2753-12.2013 PMC6119179

[36] Bullmore, E. & Sporns, O. Complex brain networks: graph theoretical analysis of structural and functional systems. *Nat. Rev. Neurosci.* **10**, 186–198 (2009). 19190637 10.1038/nrn2575

[37] Tsunoda, K., Yamane, Y., Nishizaki, M. & Tanifuji, M. Complex objects are represented in macaque inferotemporal cortex by the combination of feature columns. *Nat. Neurosci.* **4**, 832–838 (2001). 11477430 10.1038/90547

[38] Tanaka, K. Inferotemporal cortex and object vision. *Annu. Rev. Neurosci.* **19**, 109–139 (1996). 8833438 10.1146/annurev.ne.19.030196.000545

[39] Bao, P., She, L., McGill, M. & Tsao, D. Y. A map of object space in primate inferotemporal cortex. *Nature* **583**, 103–108 (2020). 32494012 10.1038/s41586-020-2350-5 PMC8088388

[40] Desimone, R., Albright, T. D., Gross, C. G. & Bruce, C. Stimulus-selective properties of inferior temporal neurons in the macaque. *J. Neurosci.* **4**, 2051–2062 (1984). 6470767 10.1523/JNEUROSCI.04-08-02051.1984 PMC6564959

[41] Tanaka, K., Saito, H., Fukada, Y. & Moriya, M. Coding visual images of objects in the inferotemporal cortex of the macaque monkey. *J. Neurophysiol.* **66**, 170–189 (1991). 1919665 10.1152/jn.1991.66.1.170

[42] Kobatake, E. & Tanaka, K. Neuronal selectivities to complex object features in the ventral visual pathway of the macaque cerebral cortex. *J. Neurophysiol.* **71**, 856–867 (1994). 8201425 10.1152/jn.1994.71.3.856

[43] Wachsmuth, E., Oram, M. W. & Perrett, D. I. Recognition of objects and their component parts: responses of single units in the temporal cortex of the macaque. *Cereb. Cortex* **4**, 509–522 (1994). 7833652 10.1093/cercor/4.5.509

[44] de Beeck, H. O. & Wagemans, J. Visual object categorisation at distinct levels of abstraction: a new stimulus set. *Perception* **30**, 1337–1361 (2001). 11768488 10.1068/p3120

[45] Elston, G. N. & Rosa, M. G. Pyramidal cells, patches, and cortical columns: a comparative study of infragranular neurons in TEO, TE, and the superior temporal polysensory area of the macaque monkey. * J. Neurosci.* **20**, RC117 (2000). 11125016 10.1523/JNEUROSCI.20-24-j0003.2000 PMC6773011

[46] Elston, G. N. & Rosa, M. Morphological variation of layer III pyramidal neurones in the occipitotemporal pathway of the macaque monkey visual cortex. *Cereb. Cortex* **8**, 278–294 (1998). 10.1093/cercor/8.3.278 9617923

[47] Elston, G. N., Oga, T., Okamoto, T. & Fujita, I. Spinogenesis and pruning in the anterior ventral inferotemporal cortex of the macaque monkey: an intracellular injection study of layer III pyramidal cells. *Front. Neuroanat.* **5**, 42 (2011). 21811440 10.3389/fnana.2011.00042 PMC3143722

[48] Sala, C. & Segal, M. Dendritic spines: the locus of structural and functional plasticity. *Physiol. Rev.* **94**, 141–188 (2014). 24382885 10.1152/physrev.00012.2013

[49] Elston, G. N. Specialization of the neocortical pyramidal cell during primate evolution. *Evolution of the Nervous Systems: A Comprehensive Reference* (eds Kaas, J. H. & Preuss, T. M.) 191–242 (Academic Press (Elsevier), 2007).

[50] McInnes, L., Healy, J. & Melville, J. Umap: Uniform manifold approximation and projection for dimension reduction. Preprint at *arXiv*10.48550/arXiv.1802.03426 (2018).

[51] Webster, M. J., Bachevalier, J. & Ungerleider, L. G. Connections of inferior temporal areas TEO and TE with parietal and frontal cortex in macaque monkeys. *Cereb. Cortex* **4**, 470–483 (1994). 7530521 10.1093/cercor/4.5.470

[52] Wang, X.-J. Macroscopic gradients of synaptic excitation and inhibition in the neocortex. *Nat. Rev. Neurosci.* **21**, 169–178 (2020). 32029928 10.1038/s41583-020-0262-x PMC7334830

[53] Kanwisher, N., McDermott, J. & Chun, M. M. The fusiform face area: a module in human extrastriate cortex specialized for face perception. *J. Neurosci.* **17**, 4302–4311 (1997). 9151747 10.1523/JNEUROSCI.17-11-04302.1997 PMC6573547

[54] Downing, P. E., Jiang, Y., Shuman, M. & Kanwisher, N. A cortical area selective for visual processing of the human body. *Science* **293**, 2470–2473 (2001). 11577239 10.1126/science.1063414

[55] Nasr, S. et al. Scene-selective cortical regions in human and nonhuman primates. *J. Neurosci.* **31**, 13771–13785 (2011). 21957240 10.1523/JNEUROSCI.2792-11.2011 PMC3489186

[56] Epstein, R. & Kanwisher, N. A cortical representation of the local visual environment. *Nature* **392**, 598–601 (1998). 9560155 10.1038/33402

[57] Weiner, K. S. & Grill-Spector, K. Neural representations of faces and limbs neighbor in human high-level visual cortex: evidence for a new organization principle. *Psychol. Res.* **77**, 74–97 (2013). 22139022 10.1007/s00426-011-0392-x PMC3535411

[58] Tong, F., Nakayama, K., Moscovitch, M., Weinrib, O. & Kanwisher, N. Response properties of the human fusiform face area. *Cogn. Neuropsychol.* **17**, 257–280 (2000). 20945183 10.1080/026432900380607

[59] Zhang, Y., Zhou, K., Bao, P. & Liu, J. A biologically inspired computational model of human ventral temporal cortex. *Neural Netw.* **178**, 106437 (2024). 10.1016/j.neunet.2024.106437 38936111

[60] Krizhevsky, A., Sutskever, I. & Hinton, G. E. Imagenet classification with deep convolutional neural networks. In *Proc. Advances in Neural Information Processing Systems* Vol. 25, 1097–1105 (Neural Information Processing Systems Foundation, Inc.; Curran Associates, Inc., 2012).

[61] Van Essen, D. C. et al. The WU-Minn human connectome project: an overview. *Neuroimage* **80**, 62–79 (2013). 23684880 10.1016/j.neuroimage.2013.05.041 PMC3724347

[62] Kubilius, J. et al. Brain-like object recognition with high-performing shallow recurrent ANNs. In *Proc. Advances in Neural Information Processing Systems* Vol. 32 (Neural Information Processing Systems Foundation, Inc.; Curran Associates, Inc., 2019).

[63] Bullmore, E. & Sporns, O. The economy of brain network organization. *Nat. Rev. Neurosci.* **13**, 336–349 (2012). 22498897 10.1038/nrn3214

[64] y Cajal, S. R. *Histology of the Nervous System of Man and Vertebrates: General Principles, Spinal Cord, Spinal Ganglia, Medulla & Pons* Vol. 1 (Oxford University Press, 1995).

[65] Theodoni, P. et al. Structural attributes and principles of the neocortical connectome in the marmoset monkey. *Cereb. Cortex* **32**, 15–28 (2022). 10.1093/cercor/bhab191 PMC8634603 34274966

[66] Ercsey-Ravasz, M. et al. A predictive network model of cerebral cortical connectivity based on a distance rule. *Neuron* **80**, 184–197 (2013). 24094111 10.1016/j.neuron.2013.07.036 PMC3954498

[67] Horvát, S. et al. Spatial embedding and wiring cost constrain the functional layout of the cortical network of rodents and primates. *PLoS Biol.* **14**, e1002512 (2016). 27441598 10.1371/journal.pbio.1002512 PMC4956175

[68] Avants, B. B., Epstein, C. L., Grossman, M. & Gee, J. C. Symmetric diffeomorphic image registration with cross-correlation: evaluating automated labeling of elderly and neurodegenerative brain. *Med. image Anal.* **12**, 26–41 (2008). 17659998 10.1016/j.media.2007.06.004 PMC2276735

[69] Glasser, M. F. et al. A multi-modal parcellation of human cerebral cortex. *Nature* **536**, 171–178 (2016). 27437579 10.1038/nature18933 PMC4990127

[70] Hopfield, J. J. Neural networks and physical systems with emergent collective computational abilities. *Proc. Natl. Acad. Sci. USA* **79**, 2554–2558 (1982). 6953413 10.1073/pnas.79.8.2554 PMC346238

[71] Amit, D. J. & Amit, D. J. *Modeling Brain Function: The World of Attractor Neural Networks* (Cambridge University Press, 1989).

[72] Hopfield, J. J. Neurons with graded response have collective computational properties like those of two-state neurons. *Proc. Natl. Acad. Sci. USA* **81**, 3088–3092 (1984). 6587342 10.1073/pnas.81.10.3088 PMC345226

[73] Hertz, J. A. *Introduction to the Theory of Neural Computation* (CRC Press, 2018).

[74] LeCun, Y., Bengio, Y. & Hinton, G. Deep learning. *nature* **521**, 436–444 (2015). 26017442 10.1038/nature14539

[75] Schmidhuber, J. Deep learning in neural networks: an overview. *Neural Netw.* **61**, 85–117 (2015). 25462637 10.1016/j.neunet.2014.09.003

[76] Freiwald, W. A., Tsao, D. Y. & Livingstone, M. S. A face feature space in the macaque temporal lobe. *Nat. Neurosci.* **12**, 1187 (2009). 19668199 10.1038/nn.2363 PMC2819705

[77] Gerstner, W., Kistler, W. M., Naud, R. & Paninski, L. *Neuronal Dynamics: From Single Neurons to Networks and Models of Cognition* (Cambridge University Press, 2014).

[78] Izhikevich, E. M. *Dynamical Systems in Neuroscience* (MIT Press, 2007).

[79] Beggs, J. M. The criticality hypothesis: How local cortical networks might optimize information processing. *Philos. Trans. R. Soc. A Math. Phys. Eng. Sci.* **366**, 329–343 (2008). 10.1098/rsta.2007.2092 17673410

[80] Morales, G. B. & Muñoz, M. A. Optimal input representation in neural systems at the edge of chaos. *Biology* **10**, 702 (2021). 34439935 10.3390/biology10080702 PMC8389338

[81] Kinouchi, O. & Copelli, M. Optimal dynamical range of excitable networks at criticality. *Nat. Phys.* **2**, 348–351 (2006).

[82] Priesemann, V. et al. Spike avalanches in vivo suggest a driven, slightly subcritical brain state. *Front. Syst. Neurosci.* **8**, 108 (2014). 25009473 10.3389/fnsys.2014.00108 PMC4068003

[83] Haimovici, A., Tagliazucchi, E., Balenzuela, P. & Chialvo, D. R. Brain organization into resting state networks emerges at criticality on a model of the human connectome. *Phys. Rev. Lett.* **110**, 178101 (2013). 23679783 10.1103/PhysRevLett.110.178101

[84] Liu, X., Fei, X. & Liu, J. The cognitive critical brain: modulation of criticality in perception-related cortical regions. *NeuroImage* **305**, 120964 (2025). 10.1016/j.neuroimage.2024.120964 39643023

[85] Pylyshyn, Z. Is vision continuous with cognition?: The case for cognitive impenetrability of visual perception. *Behav. Brain Sci.* **22**, 341–365 (1999). 11301517 10.1017/s0140525x99002022

[86] Hubel, D. H. & Wiesel, T. N. Receptive fields, binocular interaction and functional architecture in the cat’s visual cortex. * J. Physiol.* **160**, 106 (1962). 14449617 10.1113/jphysiol.1962.sp006837 PMC1359523

[87] Grill-Spector, K. & Malach, R. The human visual cortex. *Annu. Rev. Neurosci.* **27**, 649–677 (2004). 15217346 10.1146/annurev.neuro.27.070203.144220

[88] Blakemore, C. & Tobin, E. A. Lateral inhibition between orientation detectors in the cat’s visual cortex. *Exp. Brain Res.* **15**, 439–440 (1972). 5079475 10.1007/BF00234129

[89] Pinto, D. J., Brumberg, J. C., Simons, D. J., Ermentrout, G. B. & Traub, R. A quantitative population model of whisker barrels: re-examining the Wilson-Cowan equations. *J. Comput. Neurosci.* **3**, 247–264 (1996). 8872703 10.1007/BF00161134

[90] Freeman, W. J. The Hebbian paradigm reintegrated: local reverberations as internal representations. *Behav. Brain Sci.* **18**, 631–631 (1995).

[91] Douglas, R. J. & Martin, K. A. Neuronal circuits of the neocortex. *Annu. Rev. Neurosci.* **27**, 419–451 (2004). 15217339 10.1146/annurev.neuro.27.070203.144152

[92] Bi, G. -q & Poo, M. -m Synaptic modifications in cultured hippocampal neurons: dependence on spike timing, synaptic strength, and postsynaptic cell type. *J. Neurosci.* **18**, 10464–10472 (1998). 9852584 10.1523/JNEUROSCI.18-24-10464.1998 PMC6793365

[93] Markram, H., Lübke, J., Frotscher, M. & Sakmann, B. Regulation of synaptic efficacy by coincidence of postsynaptic APs and EPSPs. *Science* **275**, 213–215 (1997). 8985014 10.1126/science.275.5297.213

[94] Rigotti, M. et al. The importance of mixed selectivity in complex cognitive tasks. *Nature* **497**, 585–590 (2013). 23685452 10.1038/nature12160 PMC4412347

[95] Ma, H., Jiang, L., Liu, T. & Liu, J. From sensory to perceptual manifolds: the twist of neural geometry. *Sci. Adv.* **11**, eadv0431 (2025). 10.1126/sciadv.adv0431 PMC12693994 41370376

[96] Kim, J. H., Fiete, I. & Schwab, D. J. Superlinear precision and memory in simple population codes. Preprint at *arXiv*10.48550/arXiv.2008.00629 (2020).

[97] Kriegeskorte, N. & Wei, X.-X. Neural tuning and representational geometry. *Nat. Rev. Neurosci.* **22**, 703–718 (2021). 34522043 10.1038/s41583-021-00502-3

[98] De, A. & Chaudhuri, R. Common population codes produce extremely nonlinear neural manifolds. *Proc. Natl. Acad. Sci. USA* **120**, e2305853120 (2023). 37733742 10.1073/pnas.2305853120 PMC10523500

[99] Fusi, S., Miller, E. K. & Rigotti, M. Why neurons mix: high dimensionality for higher cognition. *Curr. Opin. Neurobiol.* **37**, 66–74 (2016). 26851755 10.1016/j.conb.2016.01.010

[100] Panzeri, S., Macke, J. H., Gross, J. & Kayser, C. Neural population coding: combining insights from microscopic and mass signals. *Trends Cogn. Sci.* **19**, 162–172 (2015). 25670005 10.1016/j.tics.2015.01.002 PMC4379382

[101] Feldman, D. E. The spike-timing dependence of plasticity. *Neuron* **75**, 556–571 (2012). 22920249 10.1016/j.neuron.2012.08.001 PMC3431193

[102] Hebb, D. O. *The Organization of Behavior: A Neuropsychological Theory* (Psychology Press, 2005).

[103] Marr, D., Willshaw, D. & McNaughton, B. *Simple Memory: A Theory for Archicortex* (Springer, 1991).

[104] Kanitscheider, I. & Fiete, I. Training recurrent networks to generate hypotheses about how the brain solves hard navigation problems. In *Proc. Advances in Neural Information Processing Systems* Vol. 30 (Neural Information Processing Systems Foundation, Inc.; Curran Associates, Inc., 2017).

[105] Marshel, J. H. et al. Cortical layer–specific critical dynamics triggering perception. *Science* **365**, eaaw5202 (2019). 31320556 10.1126/science.aaw5202 PMC6711485

[106] Isaacson, J. S. & Scanziani, M. How inhibition shapes cortical activity. *Neuron* **72**, 231–243 (2011). 22017986 10.1016/j.neuron.2011.09.027 PMC3236361

[107] Hennequin, G., Agnes, E. J. & Vogels, T. P. Inhibitory plasticity: balance, control, and codependence. *Annu. Rev. Neurosci.* **40**, 557–579 (2017). 28598717 10.1146/annurev-neuro-072116-031005

[108] Grill-Spector, K., Weiner, K. S., Kay, K. & Gomez, J. The functional neuroanatomy of human face perception. *Annu. Rev. Vis. Sci.* **3**, 167–196 (2017). 28715955 10.1146/annurev-vision-102016-061214 PMC6345578

[109] de Almeida, L., Idiart, M. & Lisman, J. E. The input–output transformation of the hippocampal granule cells: from grid cells to place fields. *J. Neurosci.* **29**, 7504–7512 (2009). 19515918 10.1523/JNEUROSCI.6048-08.2009 PMC2747669

[110] de Almeida, L., Idiart, M. & Lisman, J. E. A second function of gamma frequency oscillations: an E%-max winner-take-all mechanism selects which cells fire. *J. Neurosci.* **29**, 7497–7503 (2009). 19515917 10.1523/JNEUROSCI.6044-08.2009 PMC2758634

[111] Lisman, J. Gamma frequency feedback inhibition accounts for key aspects of orientation selectivity in V1. *Netw.: Comput. Neural Syst.* **25**, 63–71 (2014). 10.3109/0954898X.2013.877611 PMC4243463 24571098

[112] Deng, J. et al. ImageNet: A Large-Scale Hierarchical Image Database. In Proc. *2009 IEEE Conference on Computer Vision and Pattern Recognition* 248–255 (IEEE, 2009)

[113] Huang, G. B., Mattar, M., Berg, T. & Learned-Miller, E. Labeled faces in the wild: A database forstudying face recognition in unconstrained environments. In *Proc. Workshop on faces in 'Real-Life' Images: detection, alignment, and recognition* (2008).

[114] Fortunato, S. & Hric, D. Community detection in networks: a user guide. *Phys. Rep.* **659**, 1–44 (2016).

[115] Wen, H. et al. Neural encoding and decoding with deep learning for dynamic natural vision. *Cereb. Cortex* **28**, 4136–4160 (2018). 29059288 10.1093/cercor/bhx268 PMC6215471

[116] Learned-Miller, E., Huang, G. B., RoyChowdhury, A., Li, H. & Hua, G. in *Advances in Face Detection and Facial Image Analysis* 189–248 (Springer, 2016).

[117] Griffin, G., Holub, A. & Perona, P. Caltech-256 Object Category Dataset. Technical Report / Dataset release (California Institute of Technology Technical Report 7694) (California Institute of Technology, 2007).

[118] Parkhi, O. M., Vedaldi, A. & Zisserman, A. Deep Face Recognition. *British Machine Vision Conference (BMVC) 2015* (British Machine Vision Association & Society for Pattern Recognition (BMVA), 2015).

[119] Krizhevsky, A. One weird trick for parallelizing convolutional neural networks. Preprint at *arXiv*10.48550/arXiv.1404.5997 (2014).

[120] Tang, H. et al. Recurrent computations for visual pattern completion. *Proc. Natl. Acad. Sci.* **115**, 8835–8840 (2018). 30104363 10.1073/pnas.1719397115 PMC6126774

[121] Kardar, M. *Statistical Physics of Particles* (Cambridge University Press, 2007).

[122] Landau, D. & Binder, K. *A Guide to Monte Carlo Simulations in Statistical Physics* (Cambridge University Press, 2021).

[123] Bar, M. et al. Top-down facilitation of visual recognition. *Proc. Natl. Acad. Sci. USA* **103**, 449–454 (2006). 16407167 10.1073/pnas.0507062103 PMC1326160

[124] Tang, A. C. et al. Top-down versus bottom-up processing in the human brain: distinct directional influences revealed by integrating SOBI and Granger causality. In *Proc. Independent Component Analysis and Signal Separation: 7th International Conference, ICA 2007, London, UK, September 9-12, 2007* Vol. 7, 802–809 (Springer, 2007).

[125] Wykowska, A. & Schubö, A. On the temporal relation of top–down and bottom-up mechanisms during guidance of attention. *J. Cogn. Neurosci.* **22**, 640–654 (2010). 19309292 10.1162/jocn.2009.21222

