# Supplementary material for: Structured interconnectivity optimizes neural geometry for balancing specificity and generalization in object recognition
Source: Commun Biol. 2025 Dec 27;9:132. doi: 10.1038/s42003-025-09411-y (PMC12855958; doi:10.1038/s42003-025-09411-y)
Supplement: Supplementary file 1 — Supplementary Information [file 42003_2025_9411_MOESM1_ESM.pdf]

Supplementary information

	TEO	TEp	TEa
Animal	21	5	20
Vehicle	1	1	1
Face	0	2	2
Vegetable	0	1	2
House	0	4	3
Tool	0	0	2
Spiky feature	58	60	85

Table S1 Number of neurons responsive to specific object categories or features in the TEO, TEp and TEa of two macaques' IT cortex.

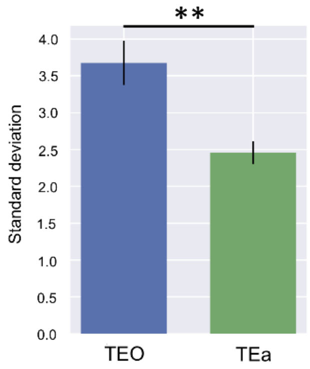

Fig. S1 Standard deviations of neuronal responses to different animal exemplars in the TEO and TEa. The standard deviation of the responses in the TEO was significantly larger than that in the TEa ($t(38) = 3.50$, $p < .01$), indicating greater variability in the TEO's response. **: $p < 0.01$.

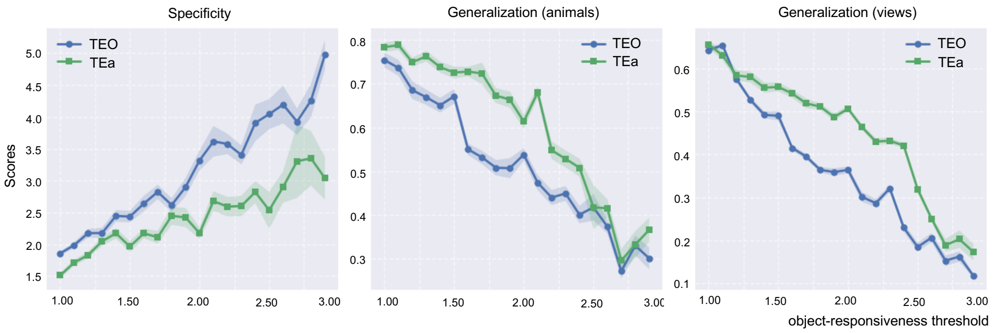

Fig. S2 Robustness of neural specificity and generalization to neuron-selection thresholds. To examine whether the observed specificity-generalization gradient depends on the object-responsiveness threshold, we systematically varied this threshold from 1.0 to 3.0 in increments of 0.1 using a grid-search sensitivity analysis. At each threshold, neurons were selected accordingly, and both specificity and generalization indices were calculated. Lines represent mean values for areas TEO (blue) and TEa (green), with shaded regions indicating 95% confidence intervals. The left panel shows specificity (animal v. non-animal), while the middle and right panels show within-category generalization across exemplars and views, respectively. Results consistently indicate robust specificity and generalization metrics across the threshold range, confirming that our primary findings are robust and independent of the neuron-selection criterion.

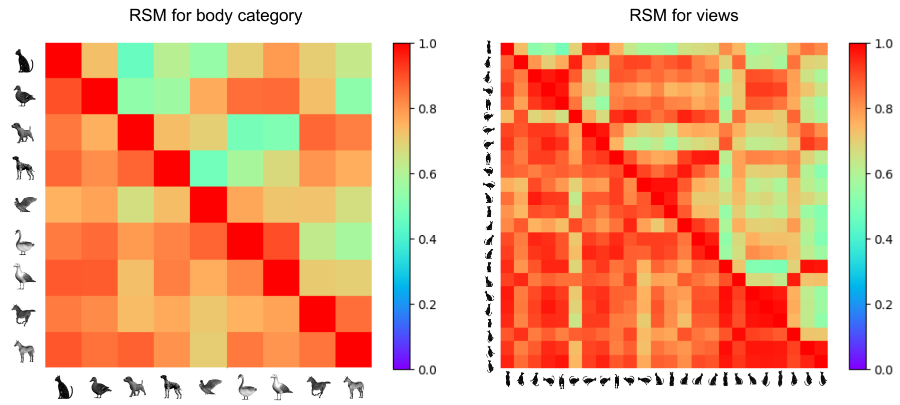

Fig. S3 Representational Similarity Matrices (RSMs) comparing population-level representations in cortical areas TEa and TEO. Left: The exemplar-level RSM is a 9×9 matrix constructed by averaging neural responses across all viewing angles for each of the nine animal exemplars. The upper triangular elements of this matrix show similarity values derived for area TEO, while the lower triangular elements correspond to area TEa. Right: the view-level RSM is a 24×24 matrix constructed by averaging neural responses across all exemplars for each of 24 distinct viewing angles. Again, the upper triangular portion reflects area TEO, and the lower triangular portion represents area TEa. Statistical analyses revealed that similarity values in the upper triangle were significantly smaller than those in the lower triangle (exemplar level: $t(38) = -13.60$, $p < .001$; view level: $t(38) = -57.42$, $p < .001$). Thus, considering both exemplar-level and view-level generalization, area TEa consistently demonstrated a higher degree of representational generalization compared to area TEO.

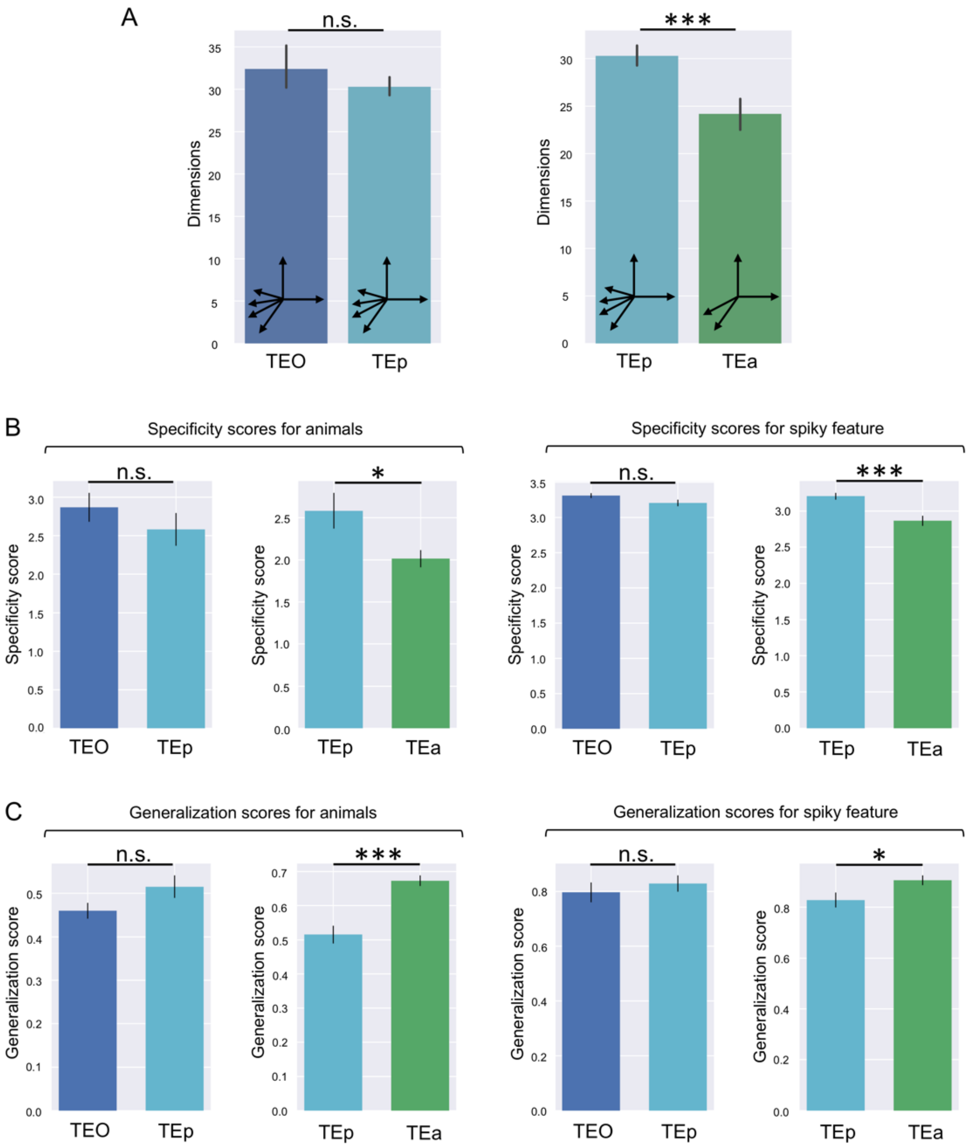

Fig. S4 Dimensionality and corresponding invariance of the TEp compared to the TEO and TEa. Note that neurons in the TEp have a similar number of dendritic spines as those in the TEO, suggesting a similar level of interconnectivity. (A) Dimensionality. The effective dimension of the TEp was not significantly different from that of the TEO ($t(38) = -1.47$, $p = .15$, top), but significantly higher than that of the TEa ($t(38) = 6.03$, $p < .001$, bottom). (B) Specificity. The specificity scores in the TEp for animals (versus the rest object categories, left in left panel) and for the spiky feature (versus the round feature, left in right panel) were comparable to those in the TEO (animal: $t(38) = 0.99$, $p = .32$; spiky: $t(38) = 1.78$, $p = .08$). While the specificity scores in the TEp for animals (right in left panel) and for spiky feature (right in right panel) were significantly higher than those in the TEa (animal: $t(38) = 2.35$, $p < .05$; spiky: $t(38) = 3.92$, p

$< .001$) (C) Generalization. The generalization scores in the TEp for animals (left in left panel) and for the spiky feature (left in right panel) were comparable to those in the TEO (animal: $t(38) = -1.71$, $p = .10$; spiky: $t(38) = -0.75$, $p = .46$). Conversely, the generalization scores in the TEp for animal exemplars (right in left panel) and for spiky stimuli (right in right panel) were significantly lower than those in the TEa (animal: $t(38) = -5.06$, $p < .001$; spiky: $t(38) = -2.28$, $p < .05$). ***: $p < 0.001$; *: $p < 0.05$.

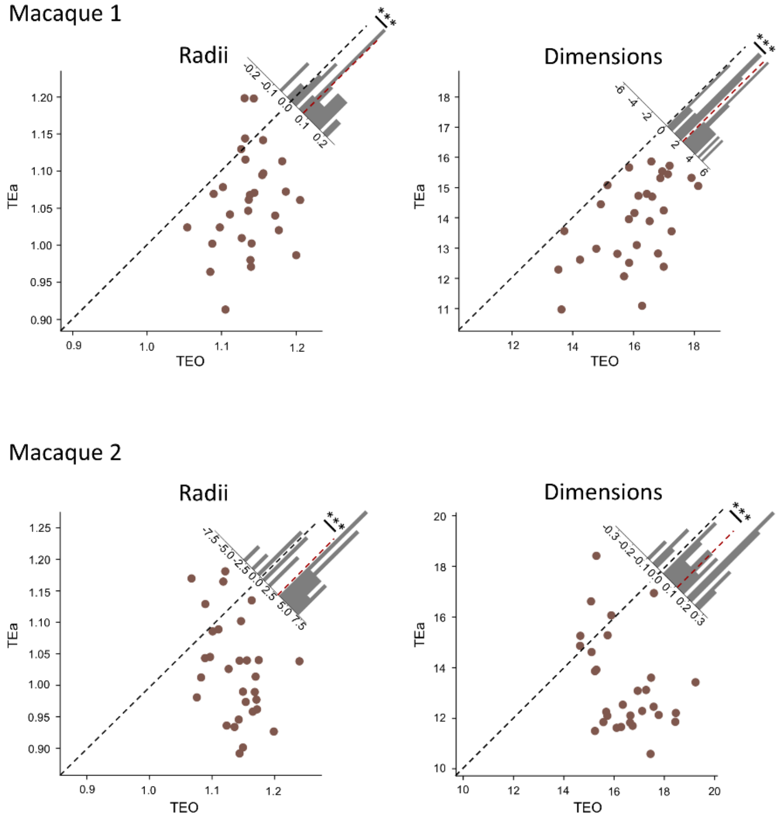

Fig. S5 Scatterplot of TEa and TEO in two macaques, analyzed separately, with each point representing the radii (left) or dimensions (right). Points below the diagonal indicate that the dimension (or radius) of the TEO was greater than that of the TEa. The dimensions in the TEa were significantly lower than those of the TEO (macaque 1: $t(58) = 6.41$, $p < .001$; macaque 2: $t(58) = 7.59$, $p < .001$), as well as radii (macaque 1: $t(58) = 5.64$, $p < .001$; macaque 2: $t(58) = 7.03$, $p < .001$). The histogram in the upper right shows the distribution of the difference between the TEO and TEa. ***: $p < 0.001$.

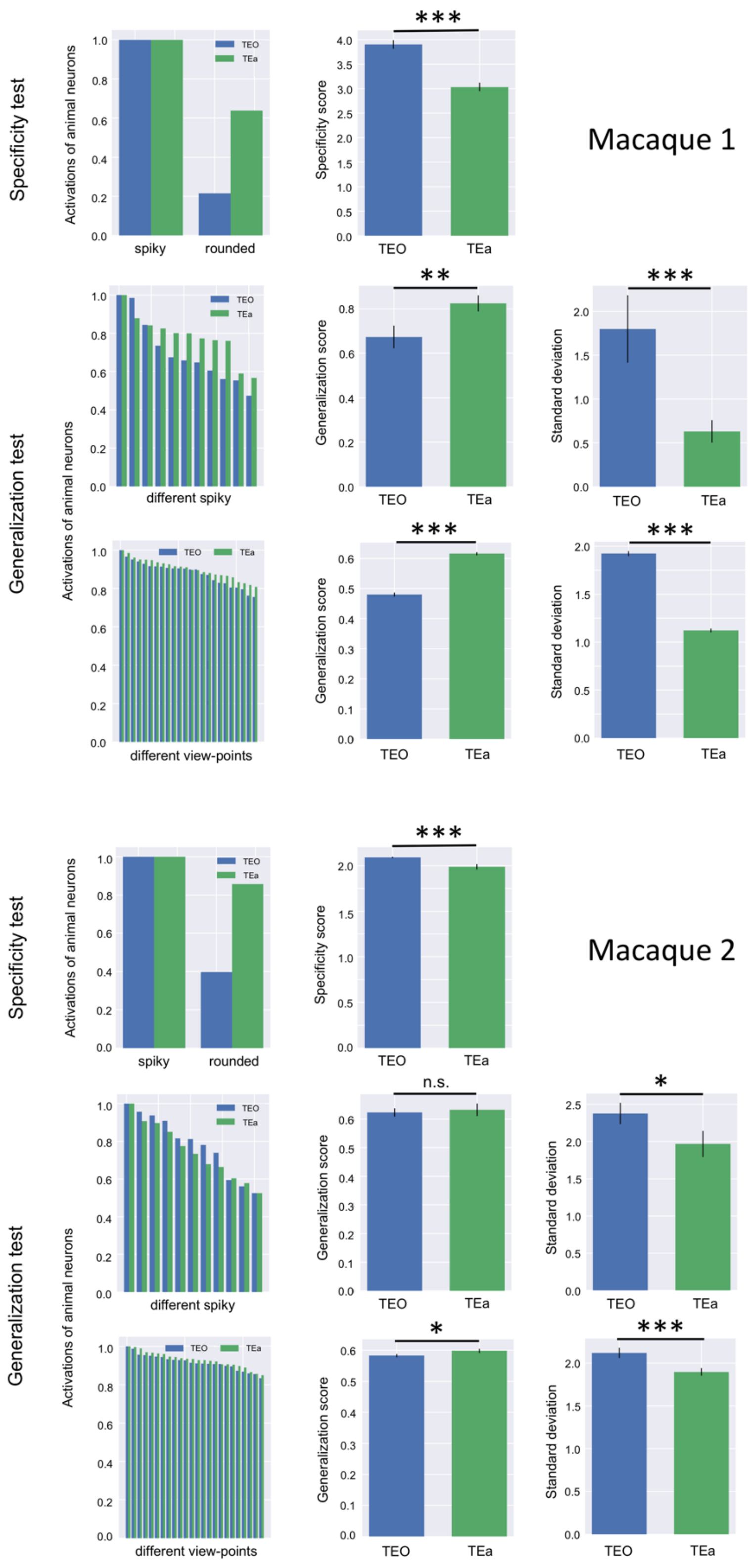

Fig. S6 Specificity and generalization in two macaques, analyzed separately. To demonstrate the consistency of the main results across the two macaques, we conducted separate tests on the specificity and generalization of spiky-responsive neurons in each monkey. Spiky features were chosen because they are abstract and activated a sufficient number of responsive neurons

necessary for the tests (Macaque1, TEO: 34, TEa: 49; Macaque2, TEO: 24, TEa: 36). In each macaque, the top row represents the specificity test. The left panel shows the mean activation of spiky-responsive neurons in the TEO and TEa in response to spiky and non-spiky (i.e., rounded) stimuli. The right panel shows that the specificity score of the TEO was significantly higher than that of the TEa (Macaque1: $t(38) = 16.71$, $p < .001$; Macaque2: $t(38) = 5.05$, $p < .001$). The middle row shows the generalization test. The left panel presents sorted mean activations of animal responsive neurons in the TEO and TEa. The middle and right panels display the generalization scores and standard deviations in the TEO and TEa. Statistical analysis shows that the TEO had lower generalization than the TEa (Macaque1: generalization score: $t(38) = -3.18$, $p < .01$; standard deviation: $t(38) = 5.80$, $p < .001$; Macaque2: generalization score: $t(38) = -0.83$, $p = 0.41$; standard deviation: $t(38) = 2.11$, $p < .05$). The bottom row replicates the middle row with the test on generalization across views of the same exemplar. Statistical analysis shows that the TEO has significantly lower generalization across views than the TEa (Macaque1: generalization score: $t(38) = -20.07$, $p < .001$; standard deviation: $t(38) = 27.20$, $p < .001$; Macaque2: generalization score: $t(38) = -2.24$, $p < .05$; standard deviation: $t(38) = -3.58$, $p < .001$). ***: $p < 0.001$; **: $p < 0.01$; *: $p < 0.05$.

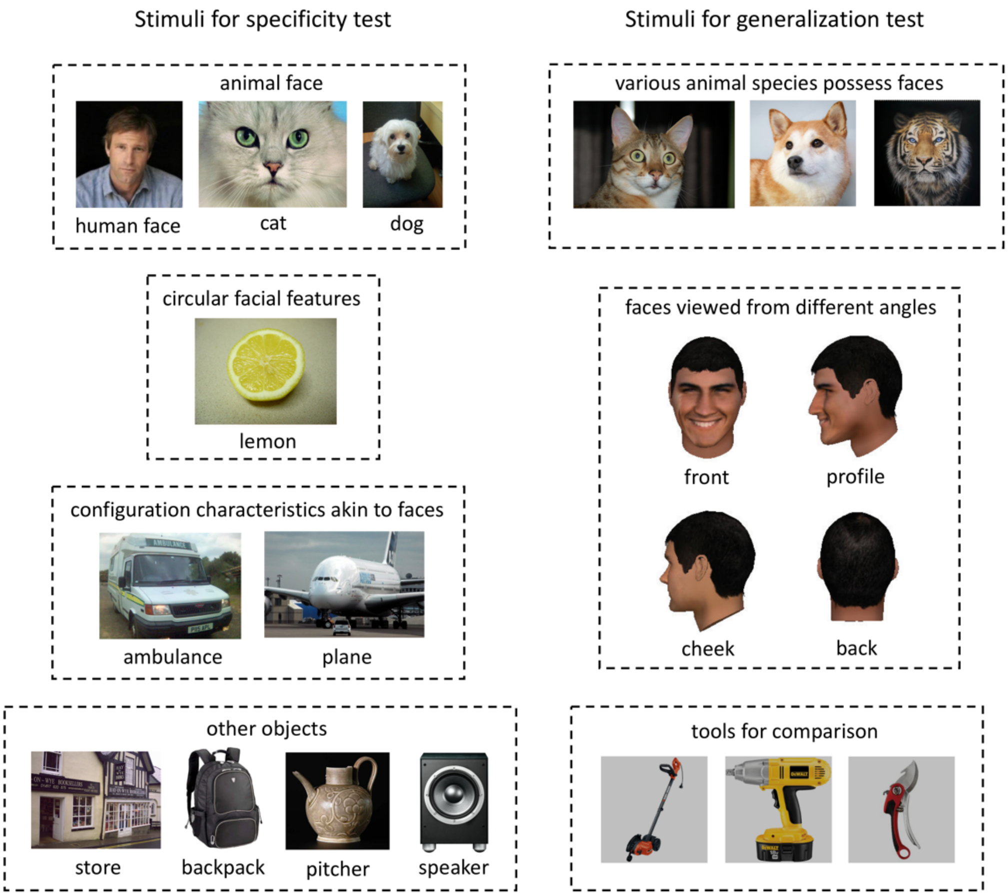

Fig. S7 Stimulus exemplars for specificity and generalization tests.

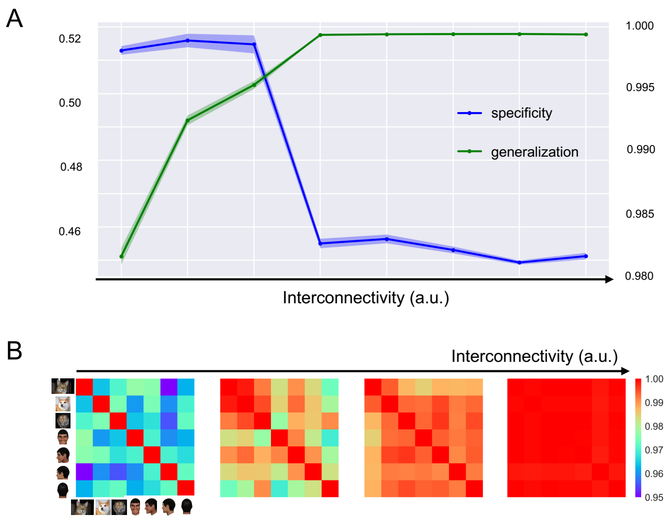

Fig. S8 Effects of interconnectivity represented by wiring length parameter (λ) on representational specificity and generalization in computational models. (A) Specificity RSA: For each λ (including 0.100, 0.075, 0.050, 0.023, 0.015, 0.010, 0.005 and 0.000), RSMs were computed using neural responses from model's face-selective units to specificity test stimuli. Each iteration used responses from 200 randomly sampled face-selective neurons. The left side of the y-axis shows the specificity scores, and the right side shows the generalization scores. The specificity score was calculated by subtracting the RSA mean normalized to 0–1 from 1, while the generalization score was simply the RSA mean normalized to 0–1. Statistical analysis revealed that mean representational similarity between face and non-face categories increased significantly as interconnectivity increased (λ decreased) (one-way ANOVA, $F(7,152) = 407.45$, $p < .001$; linear trend analysis, $F(1,152) = 446.70$, $p < .001$), indicating that narrower spatial wiring (i.e., higher λ values) leads to enhanced representational specificity. Generalization RSA: Using generalization test stimuli, mean representational similarity increased significantly as λ decreased (one-way ANOVA, $F(7,152) = 294.21$, $p < .001$; linear trend analysis, $F(1,152) = 274.17$, $p < .001$), indicating that broader interconnectivity (i.e., lower λ values) improves representational generalization across face exemplars. (B) RSM heatmap for generalization tests at different λ values (from left to right, these RSMs correspond to λ of 0.100, 0.075, 0.050 and 0.023), visually confirming that higher λ values (i.e., narrower connectivity) yielded decreased within-face similarity and greater exemplar distinctness, whereas lower λ values (i.e., broader connectivity) support more uniform responses and increased representational invariance.

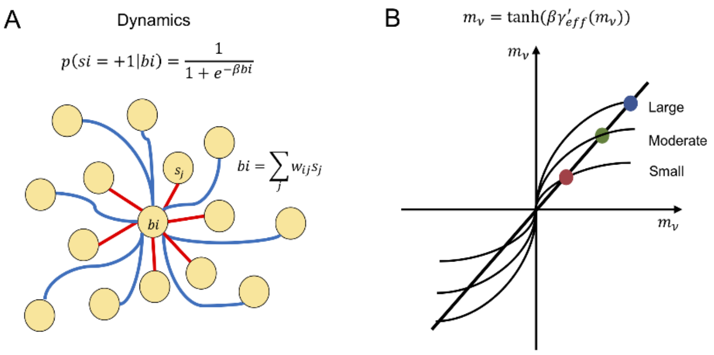

Fig. S9 The dynamics of the model and steady states in the mean-field equation. (A) The state of unit i becomes '+1' with a conditional probability $p(s_i = +1|b_i) = 1/(1 + e^{-\beta b_i})$. This probability function is sigmoid shaped, representing the likelihood that unit i will be activated under condition b_i. The magnitude of β captures the intrinsic noise in the dynamics of the system. As β increases, indicating lower noise levels, the sigmoid function becomes steeper. For our model, we set β to 100, representing a very low-level of noise, ensuring that the dynamics are driven primarily by the input conditions rather than random fluctuations. This dynamical procedure is then repeated, leading the representation pattern of model to changes dynamically over time. (B) In our model, β is fixed, while the magnitude of γ'_{eff}, representing the interconnectivity, determines the steepness of the curve associated with the model's steady state. As interconnectivity increases, the value of m_μ in the steady state also increases. A larger m_μ indicates a greater similarity to a specific pattern, leading to fewer possible states the network can occupy. For example, if $m_{face} = 1$, only one network state exists. This corresponds to a narrower attractor region, where the system is more constrained in its possible configuration, reflecting a more stable and deterministic behavior.

Appendix 1: formal proof on representation compression

We utilized the mean-field technique to understand mathematical principles governing how interconnectivity modifies the depth and radius of attractor regions in the energy-representation manifold. In our model, we used the classical order parameter in a Hopfield network to quantify the degree of overlap between the network's current state and a stored memory pattern:

$$m_\mu \equiv \frac{1}{N}\sum_{i}^{N} x_i^{(\mu)} \langle s_i \rangle$$

where $x_i^{(\mu)}$ represents the state of a stored memory pattern μ on unit i, $\langle s_i \rangle$ is the average state of unit i, and N is the total number of units. The network's energy can be expressed as: $H = -\frac{1}{2}\sum_{i,j} w_{ij} s_i s_j$, where the connection weights are given by: $w_{ij} = \frac{\gamma_{ij}}{N}\sum_{\mu=1}^{4} x_i^{(\mu)} x_j^{(\mu)}$. Substituting these weights into the energy equation gives:

$$H = -\frac{1}{N}\sum_{\mu}\sum_{i,j} \gamma_{ij} x_i^{(\mu)} s_i x_j^{(\mu)} s_j$$

For simplicity, we replace γ_{ij} with a unified effective γ_{eff}, which is proportional to the wiring length, leading to a quadratic relationship between the energy and the order parameter:

$$H \approx -\gamma_{eff}\sum_{\mu}\left(m_\mu\right)^2$$

This indicates a clear quadratic relationship between the energy and the order parameter, particularly when the state is near an attractor mode (such as $s \approx m_{face}$), the energy is directly proportional to the square of the order parameter for that mode ($H \propto m^{face}$). Due to the correlation between energy and the order parameter, the order parameter can be used as a proxy for the system's energy. Next, we investigate the relationship between the order parameter and interconnectivity.

Using the properties of two-state networks, the expression for $\langle s_i \rangle$ is given by:

$$\langle s_i \rangle = 1 \cdot p(s_i = 1|b_i) + (-1) \cdot p(s_i = -1|b_i)$$

$$\langle s_i \rangle = \frac{1}{1+e^{-\beta b_i}} - \left(1 - \frac{1}{1+e^{-\beta b_i}}\right) = \tanh(\beta b_i)$$

Under the mean-field approximation, $b_i \approx \langle b_i \rangle$, and substituting the expression for w_{ij}, we obtain:

$$b_i \approx \sum_{j}^{N} \frac{1}{N} \sum_{\mu}^{p} \gamma_{ij} x_i^{(\mu)} x_j^{(\mu)} \langle s_j \rangle$$

$$= \sum_{\mu}^{p} \frac{1}{N} x_i^{(\mu)} \sum_{j}^{N} \gamma_{ij} x_j^{(\mu)} \langle s_j \rangle$$

To simplify the equation, we similarly define an effective γ'_{eff} in place of each γ_{ij}. The γ'_{eff} and γ_{eff} have different values but have similar properties, i.e. the larger the wiring length, the larger the γ'_{eff}. Therefore:

$$b_i \approx \sum_{\mu}^{p} \frac{1}{N} x_i^{(\mu)} \sum_{j}^{N} \gamma'_{eff} x_j^{(\mu)} \langle s_j \rangle = \sum_{\mu}^{p} \gamma'_{eff} x_i^{(\mu)} m_\mu$$

Thus, we can express the order parameter m_μ as:

$$m_\mu = \frac{1}{N} \sum_{i}^{N} x_i^{(\mu)} \tanh\,(\beta b_i)$$

$$\approx \frac{1}{N} \sum_{i}^{N} x_i^{(\mu)} \tanh \left[\beta \gamma'_{eff} \sum_{\mu}^{p} x_i^{(\mu)} m_\mu \right]$$

$$= \tanh \left[\beta \gamma'_{eff} \sum_{\mu}^{p} m_\mu \right]$$

In summary, we obtain:

$$m_\mu \approx \tanh \left[\beta \gamma'_{eff} \sum_{\mu}^{p} (m_\mu + r_\mu) \right]$$

In this equation, r_μ corresponds to the resemblance between the bottom-up signal and the μ-th pattern. When the steady state of the activation pattern is close to the νth memory pattern, the equation simplifies to $m_\nu \approx \tanh[\beta \gamma'_{eff}(m_\nu + r_\nu)]$. The properties of this equation, particularly the steady state of the network, can be understood by examining the intersections of these function curves (Fig. S9 B). In the model, β is fixed, and the magnitude of γ'_{eff}, which represents the interconnectivity, determines the steepness of the curve. As the wiring length increases, the value of m_μ

at the steady state also increases. A larger value of m_μ indicates a stronger similarity to the μ-th pattern, leading to fewer states. For example, if $m_{face} = 1$, only one network state exists. In this case, the attractor region is narrow. Conversely, a smaller value of m_μ implies more available stable states, thus corresponding to a broader attractor region.

In summary, our formal proof shows that interconnectivity plays a critical role in shaping the energy landscape of the neural network. Higher interconnectivity creates more pronounced and fewer attractor states, leading to more generalized representations. Lower interconnectivity allows for a greater diversity of stable states, supporting more selective and detailed representations. This balance between specificity and generalization is crucial for efficient neural coding in cognitive tasks such as object recognition.
