# Supplementary material for: Structured interconnectivity optimizes neural geometry for balancing specificity and generalization in object recognition
Source: Commun Biol. 2025 Dec 27;9:132. doi: 10.1038/s42003-025-09411-y (PMC12855958; doi:10.1038/s42003-025-09411-y)
Supplement: Supplementary file 2 — Reporting summary [file 42003_2025_9411_MOESM2_ESM.pdf]

nature portfolio

Corresponding author(s): Jia Liu

Last updated by author(s): Dec 4, 2025

Reporting Summary

Nature Portfolio wishes to improve the reproducibility of the work that we publish. This form provides structure for consistency and transparency in reporting. For further information on Nature Portfolio policies, see our Editorial Policies and the Editorial Policy Checklist.

Statistics

For all statistical analyses, confirm that the following items are present in the figure legend, table legend, main text, or Methods section.

n/a	Confirmed	
☐	☒	The exact sample size (n) for each experimental group/condition, given as a discrete number and unit of measurement
☐	☒	A statement on whether measurements were taken from distinct samples or whether the same sample was measured repeatedly
☐	☒	The statistical test(s) used AND whether they are one- or two-sided<br>*Only common tests should be described solely by name; describe more complex techniques in the Methods section.*
☒	☐	A description of all covariates tested
☐	☒	A description of any assumptions or corrections, such as tests of normality and adjustment for multiple comparisons
☐	☒	A full description of the statistical parameters including central tendency (e.g. means) or other basic estimates (e.g. regression coefficient) AND variation (e.g. standard deviation) or associated estimates of uncertainty (e.g. confidence intervals)
☐	☒	For null hypothesis testing, the test statistic (e.g. F, t, r) with confidence intervals, effect sizes, degrees of freedom and P value noted<br>*Give P values as exact values whenever suitable.*
☒	☐	For Bayesian analysis, information on the choice of priors and Markov chain Monte Carlo settings
☒	☐	For hierarchical and complex designs, identification of the appropriate level for tests and full reporting of outcomes
☐	☒	Estimates of effect sizes (e.g. Cohen's d, Pearson's r), indicating how they were calculated

Our web collection on statistics for biologists contains articles on many of the points above.

Software and code

Policy information about availability of computer code

Data collection	We analyzed neurons in the TEO, TEa and TEp regions of the inferior temporal (IT) cortex in macaques recorded previously by Bao et al. (2020) when the macaques passively viewed a series of objects from different categories and viewing angles.
Data analysis	All custom code has been deposited at https://github.com/sdgds/Balanced-ITC.

For manuscripts utilizing custom algorithms or software that are central to the research but not yet described in published literature, software must be made available to editors and reviewers. We strongly encourage code deposition in a community repository (e.g. GitHub). See the Nature Portfolio guidelines for submitting code & software for further information.

Data

Policy information about availability of data

All manuscripts must include a data availability statement. This statement should provide the following information, where applicable:

- Accession codes, unique identifiers, or web links for publicly available datasets
- A description of any restrictions on data availability
- For clinical datasets or third party data, please ensure that the statement adheres to our policy

Provide your data availability statement here.

Research involving human participants, their data, or biological material

Policy information about studies with human participants or human data. See also policy information about sex, gender (identity/presentation), and sexual orientation and race, ethnicity and racism.

Reporting on sex and gender	The electrophysiological data used in this study were obtained from two male rhesus macaques in Bao et al. (2020). Since our study only involved basic sensory perception, gender differences did not play a role here, so the conclusions drawn from the two male macaques do not affect the universality of our conclusions.
Reporting on race, ethnicity, or other socially relevant groupings	We did not used any socially constructed or socially relevant categorization variables in the present study.
Population characteristics	This study used macaques data and computational modeling and did not use human subjects.
Recruitment	This study used macaques data and did not involve human subjects. Therefore, subject recruitment was not involved.
Ethics oversight	The data for this study were obained from Bao et al. (2020). The experiments were performed with the approval of the Caltech Institutional Animal Care and Use Committee.

Note that full information on the approval of the study protocol must also be provided in the manuscript.

Field-specific reporting

Please select the one below that is the best fit for your research. If you are not sure, read the appropriate sections before making your selection.

☒ Life sciences ☐ Behavioural & social sciences ☐ Ecological, evolutionary & environmental sciences

For a reference copy of the document with all sections, see nature.com/documents/nr-reporting-summary-flat.pdf

Life sciences study design

All studies must disclose on these points even when the disclosure is negative.

Sample size	The data for this study were obtained from the IT cortex of two precious macaques, as published by Bao et al. (2020) in Nature. Due to their availablity and the universality of perceptual principles, the data from these two macaques are sufficient to reveal the universal principles of object recognition.
Data exclusions	We used the preprocessed data from Bao et al. (2020), so we did not exclude any data.
Replication	We provide analysis code to ensure reproducible experiments and computational model recults.
Randomization	The data were obtained from Bao et al. (2020), and the macaque experiments were designed as a within-subjects design.
Blinding	The data in this study does not address this issue.

Reporting for specific materials, systems and methods

We require information from authors about some types of materials, experimental systems and methods used in many studies. Here, indicate whether each material, system or method listed is relevant to your study. If you are not sure if a list item applies to your research, read the appropriate section before selecting a response.

Materials & experimental systems

n/a	Involved in the study	
☒	☐	Antibodies
☒	☐	Eukaryotic cell lines
☒	☐	Palaeontology and archaeology
☒	☐	Animals and other organisms
☒	☐	Clinical data
☒	☐	Dual use research of concern
☒	☐	Plants

Methods

n/a	Involved in the study	
☒	☐	ChIP-seq
☒	☐	Flow cytometry
☒	☐	MRI-based neuroimaging

Plants

Seed stocks	Not applicable.
Novel plant genotypes	Not applicable.
Authentication	Not applicable.
